# Long-term exposure to insulin and volumetric mammographic density: observational and genetic associations in the Karma study

**DOI:** 10.1186/s13058-018-1026-7

**Published:** 2018-08-09

**Authors:** Signe Borgquist, Ann H. Rosendahl, Kamila Czene, Nirmala Bhoo-Pathy, Mozhgan Dorkhan, Per Hall, Judith S. Brand

**Affiliations:** 1 Division of Oncology and Pathology, Clinical Sciences, Lund University, SE-221 85 Lund, Sweden; 2 Clinical Trial Unit, Skåne University Hospital, Lund, Sweden; 3 Department of Medical Epidemiology and Biostatistics, Karolinska Institute, Solna, Sweden; 4 Julius Centre University of Malaya (JCUM), Faculty of Medicine, University of Malaya, Kuala Lumpur, Malaysia; 5 Global Medical Affairs, Novo Nordisk A/S, Søborg, Denmark; 6 Institution for Clinical Sciences, Lund University, Lund, Sweden; 7 Department of Oncology, Södersjukhuset, Stockholm, Sweden; 8 Clinical Epidemiology and Biostatistics, School of Medical Sciences, Örebro University, Örebro, Sweden

**Keywords:** Breast cancer, Insulin, Mammographic density, Diabetes, Insulin genetic score

## Abstract

**Background:**

Long-term insulin exposure has been implicated in breast cancer etiology, but epidemiological evidence remains inconclusive. The aims of this study were to investigate the association of insulin therapy with mammographic density (MD) as an intermediate phenotype for breast cancer and to assess associations with long-term elevated circulating insulin levels using a genetic score comprising 18 insulin-associated variants.

**Methods:**

We used data from the KARolinska MAmmography (Karma) project, a Swedish mammography screening cohort. Insulin-treated patients with type 1 (T1D, *n* = 122) and type 2 (T2D, *n* = 237) diabetes were identified through linkage with the Prescribed Drug Register and age-matched to 1771 women without diabetes. We assessed associations with treatment duration and insulin glargine use, and we further examined MD differences using non-insulin-treated T2D patients as an active comparator. MD was measured using a fully automated volumetric method, and analyses were adjusted for multiple potential confounders. Associations with the insulin genetic score were assessed in 9437 study participants without diabetes.

**Results:**

Compared with age-matched women without diabetes, insulin-treated T1D patients had greater percent dense (8.7% vs. 11.4%) and absolute dense volumes (59.7 vs. 64.7 cm^3^), and a smaller absolute nondense volume (615 vs. 491 cm^3^). Similar associations were observed for insulin-treated T2D, and estimates were not materially different in analyses comparing insulin-treated T2D patients with T2D patients receiving noninsulin glucose-lowering medication. In both T1D and T2D, the magnitude of the association with the absolute dense volume was highest for long-term insulin therapy (≥ 5 years) and the long-acting insulin analog glargine. No consistent evidence of differential associations by insulin treatment duration or type was found for percent dense and absolute nondense volumes. Genetically predicted insulin levels were positively associated with percent dense and absolute dense volumes, but not with the absolute nondense volume (percentage difference [95% CI] per 1-SD increase in insulin genetic score = 0.8 [0.0; 1.6], 0.9 [0.1; 1.8], and 0.1 [− 0.8; 0.9], respectively).

**Conclusions:**

The consistency in direction of association for insulin treatment and the insulin genetic score with the absolute dense volume suggest a causal influence of long-term increased insulin exposure on mammographic dense breast tissue.

**Electronic supplementary material:**

The online version of this article (10.1186/s13058-018-1026-7) contains supplementary material, which is available to authorized users.

## Background

The role of insulin in breast cancer etiology has received growing attention in recent years [1]. Basic research suggests that long-term exposure to elevated exogenous and endogenous insulins promotes breast tumor growth, either directly by signaling mitogenic effects through the insulin receptor isoform A and the insulin-like growth factor 1 (IGF-1) receptor [2, 3] or indirectly by altering the levels of circulating estrogens [4]. The potential carcinogenic effect of insulin has been demonstrated in vitro in terms of increased proliferation in human breast epithelial cells and breast cancer cell lines [2, 3]. Whether these in vitro observations are relevant to humans and concerns surrounding risks of long-term exogenous insulin use and elevated circulating insulin levels are justified remains uncertain [5–8].

Thus far, observational studies [6, 9] and randomized clinical trials [10–12] have found no compelling evidence of an increased breast cancer incidence in insulin-treated diabetes patients, although recently some studies have suggested a possible elevated risk with long-term insulin glargine use [5, 13, 14], which might be related to the more constant pharmacokinetic profile and enhanced IGF-1 receptor affinity of this insulin analog [15, 16]. However, because of methodological limitations including short-term follow-up and confounding by indication, definitive conclusions cannot be drawn. Many agents affecting carcinogenesis have long latencies and require a minimum length of exposure. Most studies addressing recency or duration effects had limited control for confounding factors or were insufficiently powered to assess associations with long-term insulin therapy [6]. While randomized clinical trials are limited in size and follow-up to study cancer-specific outcomes, confounding by indication is a concern in observational studies, and the use of different comparators is necessary to disentangle treatment effects from their underlying indications. Studies investigating associations of circulating insulin levels with breast cancer risk have also yielded conflicting results, with either positive [17–19] or null associations [20]. Most of these findings, however, were based on small numbers of breast cancer patients and a single insulin measurement, which is not an ideal proxy for long-term insulin exposure 18].

Mammographic density (MD) refers to the amount of radiologically dense fibroglandular tissue in the breast, and high MD levels are a strong and independent predictor of breast cancer risk [21, 22]. Both traits also have several reproductive and lifestyle determinants in common, and MD is viewed as an intermediate phenotype in breast cancer etiology [23]. Because many insulin-treated diabetes patients are below the age at which breast cancer is usually diagnosed, and given the long latency of breast cancer, MD serves as an attractive intermediate endpoint for identifying potential carcinogenic effects.

In the present study, we aimed to investigate the association of long-term insulin exposure with MD in a mammography screening cohort using different methodological approaches. First, we assessed associations of insulin therapy with MD by comparing insulin-treated type 1 (T1D) and type 2 (T2D) diabetes patients with age-matched individuals without diabetes, overall and stratified by duration of insulin treatment and insulin glargine use. Potential confounding by indication was addressed in case-only analyses and additional analyses comparing insulin-treated diabetes patients with patients receiving other glucose-lowering medication. Second, as an alternative means of overcoming confounding, we explored associations with an insulin genetic score in nondiabetic women. This score comprising 18 insulin-associated variants represents genetic predisposition to elevated circulating insulin levels over the life course. Because genotypes sort randomly at conception, genetic association analyses are less likely affected by confounding and hence can provide additional evidence of the likelihood of a causal effect of long-term increased insulin exposure [24].

## Methods

### Study populations

This study was nested within the KARolinska MAmmography Project for Risk Prediction of Breast Cancer (Karma), a prospective cohort of 70,877 women attending mammography screening or clinical mammography at one of four mammography units in Sweden between January 2011 and March 2013 [25]. All participants responded to a web-based questionnaire and ~ 55,000 women without a history of cancer, breast enlargement, reduction, or surgery had raw digital mammograms collected and stored at study entry, representing the study base for the present analysis. Information on diabetes diagnoses and insulin prescriptions was retrieved through linkage with the Swedish Patient Register [26] and Prescribed Drug Register [27]. The Patient Register has nationwide coverage and includes inpatient hospitalizations since 1987 and outpatient physician visits since 2001. The Prescribed Drug Register covers all drugs sold and dispensed by prescription since July 1, 2005.

Associations between insulin therapy and MD were analyzed in a matched cohort design including all insulin-treated T1D and T2D patients and a sample of age-matched individuals without diabetes. Insulin-treated diabetes patients were identified through the Prescribed Drug Register, with current insulin use defined as at least one dispensed prescription for insulin or insulin analogs (Anatomical Therapeutic Chemical Classification System [ATC] code A10A, including A10AE04 for insulin glargine) in the year prior to study entry (*see* Additional file 1: Table S1 for all identified insulin prescriptions). Diabetes diagnoses were retrieved from the Karma questionnaire and Patient Register. All register-based diagnoses were based on International Classification of Diseases (ICD) code 250 (ICD-8 and ICD-9) until 1996 and unique codes for T1D and T2D as introduced from 1997 onward (ICD-10 codes E10 and E11, respectively). Because no distinction between T1D and T2D was made in the Karma questionnaire and earlier ICD versions (ICD-8 and ICD-9), T1D and T2D patients were differentiated on the basis of previously established cutoffs of diagnosis age (T1D, ≤ 30 years; T2D, ≥ 40 years) [28, 29] when diabetes-specific codes were missing. Using these prescription and diagnostic criteria, 359 insulin-treated diabetes patients were identified, including 122 T1D and 237 T2D patients (of whom 97 T1D [79.5%] and 76 T2D [32.1%] patients were differentiated on the basis of diagnosis age). For each patient, we randomly sampled up to five individuals without a diabetes diagnosis from the study base, matched on birth year. In total, 21 T2D patients could not be matched to a maximum of 5 individuals, leaving 1771 age-matched individuals without diabetes for analyses (i.e., 610 and 1161 for insulin-treated T1D and T2D, respectively).

We further evaluated insulin treatment effects in an analysis comparing T2D patients treated with insulin only (*n* = 112) with T2D patients receiving other noninsulin glucose-lowering medication (*n* = 407). This active comparator group comprised all T2D patients with at least one dispensed prescription for glucose-lowering medication (ATC code A10B), excluding insulins in the year prior to study entry (*see* Additional file 1: Table S2 for all identified noninsulin glucose-lowering prescriptions). Because insulin is the mainstay of therapy for T1D, we were unable to do a similar analysis for this group of patients.

Finally, we examined associations with an insulin genetic score comprising 18 insulin-associated variants (*see details below*) as an instrument to proxy long-term exposure to circulating insulin levels. Associations with the insulin genetic score were assessed in a subcohort of 9437 participants with available genotyping data. All women in the subcohort had no history of cancer or diabetes at the time of study entry when blood samples were obtained. The Karma study was approved by the ethical review committee at Karolinska Institutet, and all participants provided written informed consent.

### Volumetric mammographic density

MD was estimated from raw digital mammograms collected at study entry using Volpara ™ version 1.5.0 (Volpara Solutions, Wellington, New Zealand) [30]. Volpara volumetric MD measures show good agreement with breast magnetic resonance imaging data [31] and have been validated as being predictive of breast cancer risk [30, 32]. The Volpara algorithm estimates the thickness of dense tissue at each pixel using the X-ray attenuation of an entirely fatty region as an internal reference. The absolute dense volume (cm^3^) is computed by integrating the dense thickness at each pixel over the whole mammogram, and the total breast volume (cm^3^) is derived by multiplying the breast area by the recorded breast thickness, with an appropriate correction for the breast edge. From these measures, the absolute nondense volume (cm^3^) and percentage of the breast covered by dense tissue (%) can be obtained. The average measurement of the left and right breasts of the mediolateral oblique view was taken for all analyses.

### Covariates

The following potential confounders known to be associated with MD were extracted from the baseline questionnaire: education level, body mass index (BMI, based on self-reported height and weight), lifestyle measures (smoking, alcohol intake, and physical activity), reproductive and hormonal factors (age at menarche, number of births/age at first birth, menopausal status, use of oral contraceptives and hormone replacement therapy), and personal history of previous benign breast disease and breast cancer heredity. We also extracted information on prescriptions of comedication through linkage with the Prescribed Drug Register using the ATC coding system (including low-dose aspirin [ATC code B01AC06], statins [ATC codes C10AA01, C10AA03, C10AA04, C10AA05, C10AA07, C10AA08], and metformin [ATC code A10BA02]) and summarized data on comorbid conditions derived from the Patient Register into the Charlson comorbidity index score [33].

### Insulin genetic score

Associations with the insulin genetic score were assessed in the Karma subcohort with genotyping data. Whole-blood samples of 9437 study participants were genotyped using Illumina iSelect arrays (iCOGS [*n* = 3909] and OncoArray [*n* = 5528]), details of which have been described elsewhere [34, 35], and missing genotypes for common variants across the genome were imputed using the 1000 Genomes Project March 2012 release as a reference. The insulin genetic score was constructed using 18 independent single-nucleotide polymorphisms (SNPs) shown to be robustly associated with circulating log insulin levels at *P* < 5.0 × 10^− 8^ [36] (Additional file 1: Table S3). The score was calculated on the basis of a weighted method according to each SNP’s effect size (*β*) obtained from the literature [24]:

$$ \mathrm{Insulin}\ \mathrm{genetic}\ \mathrm{score}={\beta}_1{x}_1+{\beta}_2{x}_2+.\dots \kern0.5em {\beta}_k{x}_k+{\beta}_n{x}_n, $$

where *β* is the per-allele beta value for log-transformed fasting insulin levels associated with the effect allele for SNP *k* and *x*_*k*_ is the number of alleles for the same SNP (0, 1, 2) and *n* is the total number of SNPs included in the score (*n* = 18).

### Statistical analyses

To approximate the normal distribution, all mammographic measures were log-transformed prior to analyses, and geometric means and percentage (%) differences were calculated [37]. Differences in MD by insulin therapy were analyzed using generalized linear models, including a basic model with adjustment for age and BMI and a multivariable model with inclusion of other potential confounders (education level, age at menarche, parity and age at first birth, oral contraceptives, menopausal status, hormone replacement therapy, alcohol intake, smoking status, statins, low-dose aspirin, Charlson comorbidity index, history of benign breast disease, and family history of breast cancer). Analyses were additionally adjusted for metformin comedication to account for potential antiproliferative effects of this diabetes drug [6]. Differences in MD were assessed overall and by insulin glargine use and treatment duration. In T2D patients, treatment duration was defined from the first dispensed insulin prescription encountered in the Prescribed Drug Register. Because age at T1D onset is well below the age at which women undergo mammography screening, and with prescription data being available only from July 2005 onward, insulin treatment duration in T1D patients was calculated from the age at T1D diagnosis to study entry.

To address possible residual confounding by underlying disease, we also examined MD differences comparing insulin-treated with non-insulin-treated T2D patients. This active comparator analysis was adjusted for the same covariates as listed above and additionally for diabetes duration to account for differences in disease onset between the two patient groups.

Associations with the insulin genetic score were assessed using linear regression, with beta values representing percentage differences in MD per 1-SD increment in the score. Because there was no evidence of heterogeneity by genotyping array (iCOGS vs. OncoArray), a one-sample approach was undertaken. Genetic score analyses were adjusted for age, BMI, menopausal status, genotyping array, and six principal components to account for population stratification. To investigate the independence of genetic effects of other potential confounders, we also examined associations of the score with covariates entered in the insulin treatment analysis.

All analyses were undertaken using STATA version 14 (StataCorp, College Station, TX, USA) and PLINK version 1.9 [38]. Missing values on covariates were imputed using multivariate multiple imputation with chained equations, and ten imputed datasets were generated [39]. Imputation models included the outcome (MD), exposure (insulin treatment/insulin genetic score), and all covariates included in any of the analysis models (*see* Additional file 2: Supplementary Methods).

## Results

Descriptive characteristics of the study populations are summarized in Table 1. Mean ages at diagnosis were 20 and 55 years for the insulin-treated T1D and T2D patients, respectively. Insulin glargine was prescribed to 53% of T1D patients and 31% of T2D patients, and combined therapy with metformin was given in 4% and 53% of T1D and T2D patients, respectively. Compared with age-matched individuals without diabetes, insulin-treated T1D and T2D patients were younger at first child’s birth, less often used oral contraceptives in the past, more frequently reported a family history of breast cancer, and were more often alcohol abstainers and less physically active at study entry. As expected, insulin-treated T1D and T2D patients also presented with more comorbid conditions and were more often on statin and low-dose aspirin medications. Univariate associations with other participant characteristics were comparable for the two patient groups, except for BMI and age at menarche. Study participants of the subcohort for genetic analysis had characteristics similar to those of the age-matched individuals without T2D, except for a larger proportion being premenopausal. Descriptive characteristics of insulin-treated and non-insulin-treated T2D patients are summarized in Additional file 1: Table S4. Compared with T2D patients receiving other glucose-lowering medication, insulin-treated T2D patients were younger, had a lower BMI, and were more likely to have comorbid conditions and a history of benign breast disease. They also had a longer disease duration (8.3 vs. 4.8 years), as reflected by the younger age at diagnosis.

**Table 1:** Descriptive characteristics of the study population

Characteristic	Matched cohort analysis insulin-treated diabetes						Insulin genetic score analysis
	Insulin-treated T1D (*n* = 122)	Non-diabetics (*n* = 610)	*P* value	Insulin-treated T2D (*n* = 237)	Nondiabetics (*n* = 1161)	*P* value	Nondiabetics (*n* = 9437)
Age, years, mean (SD)	49.6 (8.6)	49.6 (8.5)	1.00	62.5 (8.0)	62.3 (7.9)	0.82	57.3 (9.8)
BMI, kg/m^2^, mean (SD)	25.7 (4.3)	25.1 (4.3)	0.17	29.5 (5.7)	25.3 (3.9)	< 0.001	25.3 (4.1)
Education level, % (*n*)			0.09			0.09	
Compulsory	8.5 (10)	8.3 (49)		27.9 (62)	22.7 (250)		16.9 (1376)
Gymnasium	40.2 (47)	30.2 (178)		29.7 (66)	27.2 (300)		31.2 (2544)
University	51.3 (60)	61.5 (362)		42.3 (94)	50.1 (552)		51.9 (4234)
Missing	*4.1 (5)*	*3.4 (21)*		*6.3 (15)*	*5.1 (59)*		*13.6 (1283)*
Age at menarche, years, mean (SD)	13.4 (2.0)	13.0 (1.4)	0.02	12.8 (1.5)	13.3 (1.5)	< 0.001	13.2 (1.5)
Parity, % (*n*)			< 0.001			0.12	
0	22.1 (27)	14.0 (85)		17.9 (42)	12.6 (144)		12.1 (1129)
1	25.4 (31)	14.0 (85)		14.5 (34)	12.9 (147)		14.5 (1354)
2	36.9 (45)	51.8 (314)		42.7 (100)	46.8 (534)		47.6 (4460)
≥ 3	15.6 (19)	20.1 (122)		24.8 (58)	27.8 (317)		25.9 (2423)
Missing	*0.0 (0)*	*0.7 (4)*		*1.3 (3)*	*1.6 (19)*		*0.8 (71)*
Age at first birth, years, mean (SD)	27.3 (5.6)	28.5 (5.3)	0.05	24.3 (4.4)	25.8 (5.0)	< 0.001	26.6 (5.0)
Missing	*5.3 (5)*	*5.6 (29)*		*6.3 (12)*	*5.1 (51)*		
Menopausal status, % (*n*)			0.97			0.86	
Premenopausal	65.6 (80)	65.4 (399)		13.5 (32)	14.0 (162)		34.1(3216)
Postmenopausal	34.4 (42)	34.6 (211)		86.5 (205)	86.0 (999)		65.9 (6221)
OC use (ever), % (*n*)	73.8 (90)	85.6 (519)	0.001	66.1 (154)	75.3 (853)	0.004	79.0 (6779)
Missing	*0.0 (0)*	*0.7 (4)*		*1.7 (4)*	*2.4 (28)*		*9.1 (857)*
HRT, % (*n*)			0.23			0.52	
Never	86.8 (99)	86.9 (504)		71.2 (151)	70.6 (730)		73.6 (6400)
Former	7.9 (9)	10.5 (61)		25.5 (54)	24.3 (251)		21.9 (1909)
Current	5.3 (6)	2.6 (15)		3.3 (7)	5.1 (53)		4.8 (423)
Missing	*6.6 (8)*	*4.9 (30)*		*10.5 (25)*	*10.9 (127)*		*7.5 (705)*
Alcohol intake, % (*n*)			0.02			< 0.001	
None	27.9 (34)	17.0 (104)		44.4 (103)	17.7 (200)		18.9 (1602)
1–25 g/wk	17.2 (21)	26.2 (160)		18.5 (43)	23.2 (262)		19.3 (1643)
25–50 g/wk	32.8 (40)	27.7 (169)		16.4 (38)	29.5 (333)		32.3 (2742)
> 50 g/wk	21.3 (26)	27.9 (170)		20.7 (48)	29.5 (332)		28.5 (2508)
Missing	*0.8 (1)*	*1.1 (7)*		*2.1 (5)*	*2.9 (34)*		*10.0 (942)*
Physical activity, % (*n*)			0.07			< 0.001	
< 40 MET h/d	40.2 (47)	31.6 (188)		54.1 (120)	39.9 (443)		35.4 (2913)
40–45 MET h/d	32.5 (38)	36.8 (219)		32.0 (71)	35.4 (393)		36.0 (2969)
45–50 MET h/d	12.8 (15)	20.8 (124)		8.6 (19)	16.5 (183)		18.6 (1535)
> 50 MET h/d	14.5 (17)	10.8 (64)		5.4 (12)	8.1 (90)		10.0 (822)
Missing	*4.1 (5)*	*2.5 (15)*		*6.3 (15)*	*4.5 (52)*		*12.7 (1198)*
Smoking status, % (*n*)			0.38			0.17	
Never	52.5 (64)	53.9 (327)		41.1 (97)	46.5 (536)		46.4 (3977)
Former	32.0 (39)	34.9 (212)		44.5 (105)	42.6 (491)		41.7 (3568)
Current	15.6 (19)	11.2 (68)		14.4 (34)	10.9 (125)		11.9 (1020)
Missing	*0.0 (0)*	*0.5 (3)*		*0.4 (1)*	*0.8 (9)*		*9.2 (872)*
Statin therapy (current), % (*n*)	36.1 (44)	4.3 (26)	< 0.001	61.2 (145)	11.5 (134)	< 0.001	8.9 (839)
Low-dose aspirin (current), % (*n*)	15.6 (19)	2.1 (13)	< 0.001	35.0 (83)	6.5 (76)	< 0.001	5.1 (483)
Charlson comorbidity index, % (*n*)			< 0.001			< 0.001	
0	82.0 (100)	97.0 (592)		73.8 (175)	92.2 (1070)		94.5 (8913)
1	14.8 (18)	2.6 (16)		15.2 (36)	6.5 (75)		4.8 (452)
≥ 2	3.3 (4)	0.3 (2)		11.0 (26)	1.4 (16)		0.8 (72)
Benign breast disease, % (*n*)			0.77			0.78	
No	78.1 (89)	76.8 (464)		75.8 (175)	74.9 (856)		76.8 (7084)
Yes	21.9 (25)	23.2 (140)		24.2 (56)	25.1 (287)		23.2 (2145)
Missing	*6.6 (8)*	*1.0 (6)*		*2.5 (6)*	*1.6 (18)*		*2.2 (208)*
Family history of breast cancer, % (*n*)			0.05			0.04	
No	84.0 (100)	90.2 (534)		80.9 (182)	86.1 (963)		87.0 (7944)
Yes	16.0 (19)	9.8 (58)		19.1 (43)	13.9 (155)		13.0 (1188)
Missing	*2.5 (3)*	*3.0 (18)*		*5.1 (12)*	*3.7 (43)*		*3.2 (305)*
Age at diagnosis, years, mean (SD)	19.9 (7.6)	–		54.8 (8.2)	–		–
Diabetes duration, years, mean (SD)	29.7 (10.2)	–		4.9 (2.5)	–		–
Insulin therapy, % (*n*)							
Glargine insulin	53.3 (65)	–		31.2 (74)	–		–
Nonglargine insulin	46.7 (57)	–		68.8 (163)	–		–
Comedication metformin, % (*n*)							
No	95.9 (117)	–		47.3 (112)	–		–
Yes	4.1 (5)	–		52.7 (125)	–		–

*Abbreviations: T1D* Type 1 diabetes, *T2D* Type 2 diabetes, *BMI* Body mass index, *OC* Oral contraceptive, *HRT* Hormone replacement therapy, *MET* Metabolic equivalent of activity level

Study populations: matched cohort analysis including insulin-treated diabetes and insulin genetic score analysis including women with available genotyping data and no known diabetes. All women in the study population were free of cancer at study entry (i.e., the baseline screening visit). In total, 21 T2D patients could not be matched to a maximum of 5 individuals (i.e., 20 patients were matched to 4 individuals, and 1 patient was matched to 1 individual), leaving 610 and 1161 age-matched women without diabetes for insulin-treated T1D and T2D analyses, respectively. Participant characteristics were compared using *t* tests for continuous data and chi-squared tests for categorical variables

A summary plot of MD percentage differences estimated in each observational analysis is presented in Additional file 3: Figure S1 to facilitate comparison across the different analyses. Geometric means of MD in insulin-treated diabetics and age-matched individuals without diabetes are listed in Table 2 together with corresponding percentage differences. Compared with age-matched women without diabetes, T1D patients had a greater percent dense (11.3% vs. 8.7%) and absolute dense (65.7 vs. 59.6 cm^3^) volume and a smaller absolute nondense volume (510 vs. 610 cm^3^). These associations were not materially different after multivariable adjustment (percent dense volume [11.4 vs. 8.7%], absolute dense volume [64.7 vs. 59.7 cm^3^], and absolute nondense volume [491 vs. 615 cm^3^]). Compared with women without diabetes, the largest difference in absolute dense volume was found for current use of insulin glargine, whereas for percent dense and absolute nondense volumes, no notable difference in magnitude of association was observed by insulin type. Overall, similar associations were found for insulin-treated T2D. Compared with age-matched individuals without diabetes, insulin-treated T2D patients had greater percent dense (7.8% vs. 6.5%) and absolute dense (58.0 vs. 52.6 cm^3^) volumes, as well as a smaller absolute nondense volume (754 vs. 679 cm^3^). As for T1D, insulin glargine use was associated with the largest difference in absolute dense volume in T2D patients.

**Table 2:** Geometric means and percentage differences of volumetric mammographic density comparing insulin-treated T1D and T2D patients with age-matched individuals without diabetes

	No. of subjects	Geometric mean (95% CI)					
		Percent dense volume (%)		Absolute dense volume (cm^3^)		Absolute nondense volume (cm^3^)	
		Age- and BMI-adjusted	Multivariable-adjusted	Age- and BMI-adjusted	Multivariable-adjusted	Age- and BMI-adjusted	Multivariable-adjusted
Insulin-treated T1D							
Nondiabetics	610	8.7 (8.5; 9.1)	8.7 (8.4; 9.0)	59.6 (57.4; 61.9)	59.7 (57.5; 62.1)	610.3 (589.5; 631.8)	615.0 (593.8; 637.0)
T1D - insulin any	122	11.3 (10.4; 12.2)	11.4 (10.5; 12.4)	65.7 (60.4; 71.5)	64.7 (58.8; 71.1)	509.8 (471.8; 550.9)	490.5 (450.0; 534.6)
*P* value		< 0.001	< 0.001	0.01	0.14	< 0.001	< 0.001
Nondiabetics	610	8.7 (8.5; 9.1)	8.7 (8.4; 9.0)	59.6 (57.4; 61.9)	59.8 (57.6; 62.2)	611.0 (590.2; 632.6)	615.7 (594.5; 637.7)
T1D - nonglargine insulin	57	11.0 (9.8; 12.3)	11.5 (10.2; 13.0)	60.0 (52.7; 68.2)	59.1 (51.5; 67.9)	477.1 (423.9; 536.9)	447.2 (394.3; 507.2)
T1D - glargine insulin	65	11.5(10.4; 12.8)	11.4 (10.2; 12.7)	70.9 (63.2; 79.6)	69.2 (61.3; 78.2)	534.1 (480.3; 593.9)	525.9 (470.8; 587.4)
*P* value		< 0.001	< 0.001	0.02	0.07	< 0.001	< 0.001
Insulin-treated T2D							
Nondiabetics	1161	6.5 (6.4; 6.7)	6.5 (6.3; 6.6)	52.7 (51.3; 54.1)	52.6 (51.2; 54.0)	746.9 (728.3; 766.0)	753.5(734.5; 773.0)
T2D - insulin any	237	7.4 (6.9; 8.0)	7.8 (7.2; 8.4)	57.5 (53.5; 61.9)	58.0 (53.7; 62.7)	708.9 (661.3; 760.0)	679.1(630.9; 731.0)
*P* value		0.002	0	0.004	0.03	0.20	0.02
Nondiabetics	1161	6.5 (6.4; 6.7)	6.5 (6.3; 6.7)	52.8 (51.4; 54.3)	52.7 (51.3; 54.2)	746.6 (727.8; 765.8)	753.0 (733.9; 772.6)
T2D - nonglargine insulin	163	7.1 (6.5; 7.8)	7.5 (6.8; 8.2)	55.2 (50.4; 60.4)	56.0 (50.9; 61.5)	715.3 (656.1; 779.9)	687.1 (628.0; 751.7)
T2D – glargine insulin	74	7.9 (7.1; 8.7)	8.2 (7.4; 9.1)	60.7 (54.9; 67.1)	60.7 (54.8; 67.3)	700.8 (636.7; 771.4)	669.2 (606.6; 738.3)
*P* value		0.002	< 0.001	0.04	0.04	0.41	0.05
		Percentage difference (95% CI)					
		Percent dense volume		Absolute dense volume		Absolute nondense volume	
		Age- and BMI-adjusted	Multivariable-adjusted	Age- and BMI-adjusted	Multivariable-adjusted	Age- and BMI-adjusted	Multivariable-adjusted
Insulin-treated T1D							
Nondiabetics	610	Reference	Reference	Reference	Reference	Reference	Reference
T1D - insulin any	122	28.8 (18.3; 40.2)	31.2 (19.5; 44.0)	10.3 (0.5; 21.0)	8.2 (−2.6; 20.3)	−16.5 (−23.3; −9.0)	−20.2 (−27.6; −12.2)
Nondiabetics	610	Reference	Reference	Reference	Reference	Reference	Reference
T1D - nonglargine insulin	57	25.6 (11.2; 41.8)	31.8(15.7; 50.2)	0.6 (− 12.1; 15.2)	−1.2 (− 14.7; 14.4)	−21.9 (−31.0; − 11.6)	−27.4 (−36.5; − 17.0)
T1D - glargine insulin	65	31.3 (17.7; 46.5)	30.7 (16.5; 46.6)	19.1 (5.4; 34.5)	15.7 (1.7; 31.7)	−12.6 (− 21.8; − 2.3)	−14.6 (− 24.1; − 3.9)
Insulin-treated T2D							
Nondiabetics	1161	Reference	Reference	Reference	Reference	Reference	Reference
T2D - insulin any	237	13.7 (4.7; 23.6)	20.5 (10.3; 31.6)	9.2 (0.4; 18.7)	10.3 (0.9; 20.6)	−5.1 (−12.4; 2.8)	−9.9 (− 17.2; − 1.9)
Nondiabetics	1161	Reference	Reference	Reference	Reference	Reference	Reference
T2D - nonglargine insulin	163	8.3 (−2.1; 19.7)	15.0 (3.6; 27.7)	4.5 (− 5.5; 15.6)	6.2 (−4.5; 18.1)	−4.2 (−13.0; 5.5)	−8.8 (− 17.5; 1.0)
T2D – glargine insulin	74	20.4 (8.3; 33.8)	26.9 (13.8; 41.6)	14.9 (3.3; 27.8)	15.2 (3.1; 28.6)	−6.1 (−15.2; 3.9)	−11.1 (−20.0; −1.3)

*Abbreviations: T1D* Type 1 diabetes, *T2D* Type 2 diabetes, *BMI* Body mass index

Multivariable-adjusted model: model adjusted for age (years), body mass index (kg/m^2^), education level (compulsory, gymnasium, university), age at menarche (years), parity, and age at first birth (nulliparous, parous/age at first birth < 25 years, parous/age at first birth 25–30 years, parous/age at first birth > 30 years), menopausal status (premenopausal, postmenopausal), oral contraceptives (never, ever), hormone replacement therapy (never, former, current), alcohol intake (none, 1–25 g/wk, 25–50 g/wk, > 50 g/wk), physical activity (< 40 MET h/d, 40–45 MET h/d, 45–50 MET h/d, > 50 MET h/d), smoking status (never, former, current), statins (no, yes), low-dose aspirin (no, yes), Charlson comorbidity index (0, 1, ≥ 2), benign breast disease (no, yes), and family history of breast cancer (no, yes). All analyses were standard adjusted for metformin therapy

A positive association of insulin glargine use with the absolute dense volume was also observed in case-only analyses including insulin-treated T1D and T2D patients only (Additional file 1: Table S5), despite the lower level of statistical significance due to smaller sample size. The association of insulin glargine with the absolute nondense volume, however, was directionally inconsistent with the association observed in analyses using age-matched nondiabetics as a comparator **(**Additional file 1: Table S5, Additional file 3: Figure S1). In case-only analyses, glargine insulin users had a greater (T2D) or similar-sized (T1D) absolute nondense volume (and consequently similar-sized (T2D) or greater (T1D) percent dense volume) compared with nonglargine insulin users. Associations between insulin therapy and MD were not very different in analyses comparing insulin-treated T2D patients with T2D patients receiving noninsulin glucose-lowering medication; that is, insulin-treated T2D patients had a greater percent and absolute dense volume and a smaller absolute nondense volume than non-insulin-treated T2D patients, with the absolute dense volume association being specific for insulin glargine use (Additional file 1: Table S6).

**Fig. 1:**
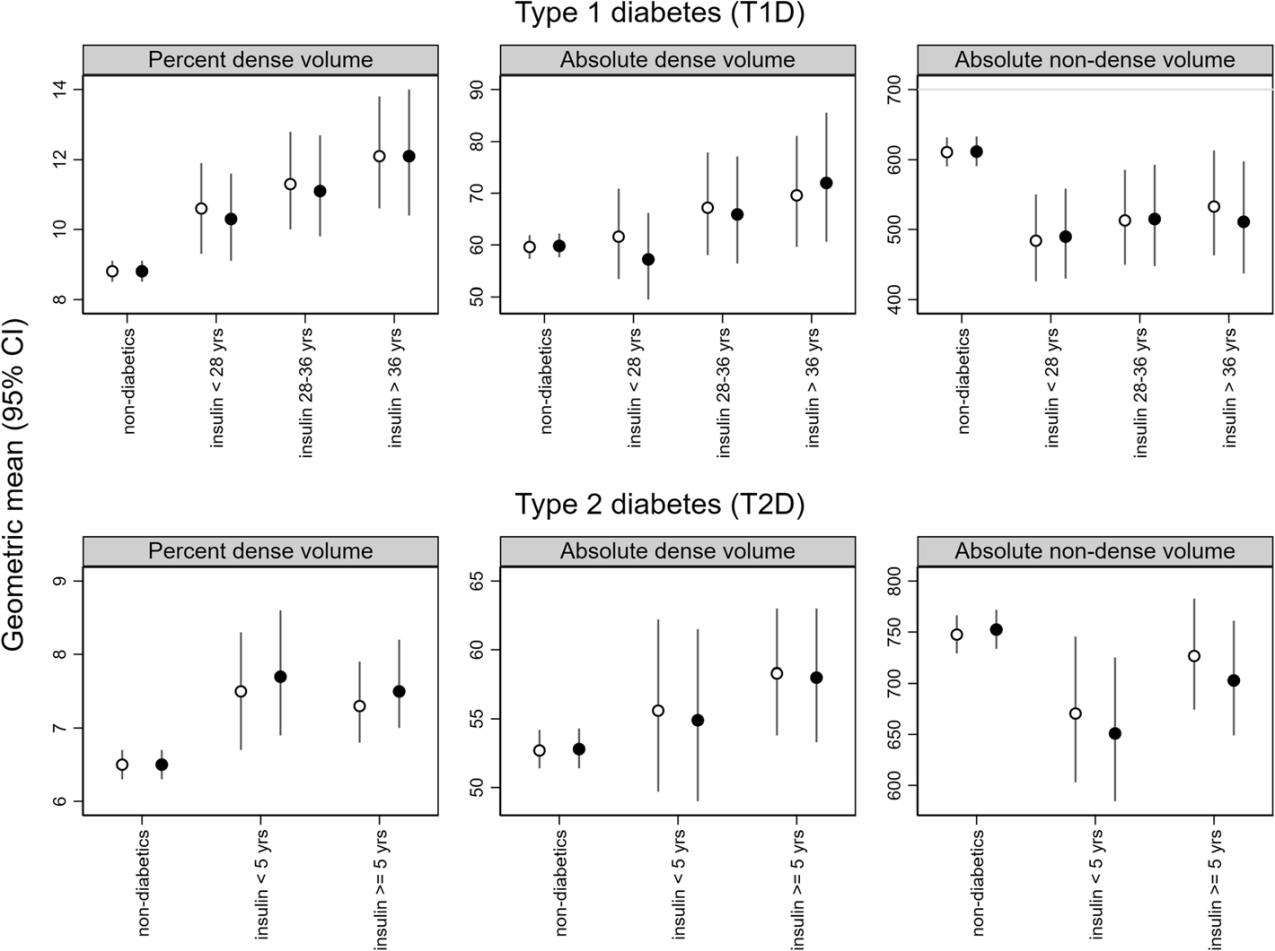
Associations of duration of insulin therapy with volumetric mammographic density in type 1 diabetes (T1D) and type 2 diabetes (T2D) patients. Geometric means and 95% CIs of volumetric mammographic density by duration of insulin therapy in T1D and T2D patients. Model 1 (*open circles*): adjusted for age and body mass index. Model 2 (*closed circles*): adjusted for age, body mass index, education level, age at menarche, parity, age at first birth, menopausal status, oral contraceptives use, hormone replacement therapy, alcohol intake, physical activity, smoking status, statins, low-dose aspirin, Charlson comorbidity index, benign breast disease, family history of breast cancer, and metformin therapy

Results of analyses by insulin treatment duration are summarized in Fig. 1 and Additional file 1: Table S7. Compared with age-matched individuals without diabetes, a gradual increase in absolute dense volume was found with duration of insulin treatment in T1D patients. In T2D patients, a similar increase in absolute dense volume was observed with treatment duration. Case-only analyses in insulin-treated T1D patients supported the presence of a treatment duration effect on the absolute dense volume (*P* trend = 0.04), though numbers were small, and the association with long-term therapy (beyond 28 years) attenuated following multivariable adjustment (*P* trend = 0.23) (Additional file 1: Table S8). No consistent evidence of a treatment duration effect was found for percent dense and absolute nondense volumes, overall and in case-only analyses (Additional file 3: Figure S1, Additional file 1: Tables S7 and S8).

**Fig. 2:**
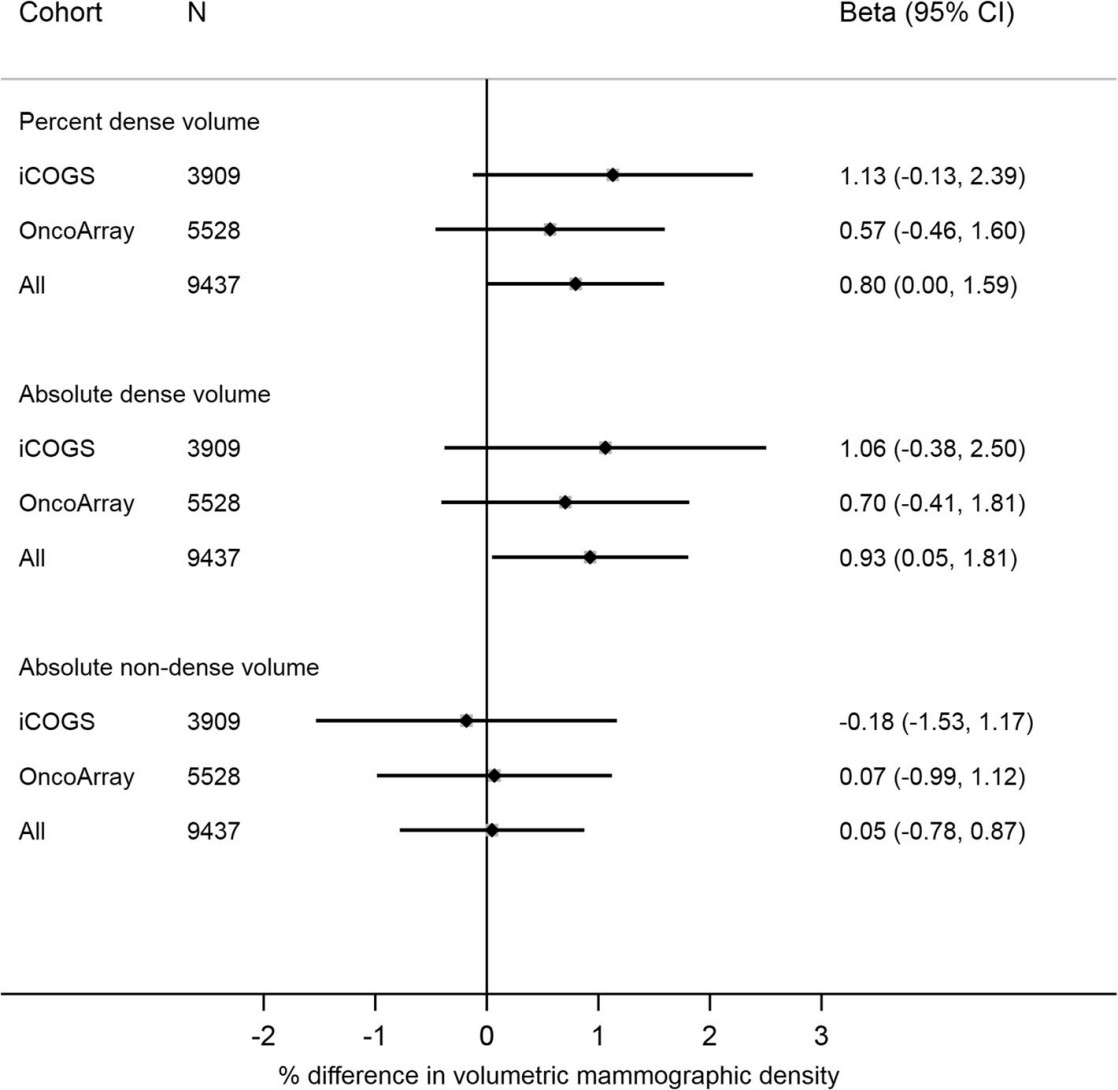
Association of the insulin genetic score with volumetric mammographic density. Association of 18-single-nucleotide polymorphism insulin genetic score with volumetric mammographic density in nondiabetic women, overall and stratified by genotyping array. Associations with volumetric mammographic density were analyzed in a linear regression model, adjusted for age, body mass index, menopausal status, and six principal components. Analyses in the total study population were additionally adjusted for genotyping array. All volumetric mammographic density measures were log-transformed prior to analyses, with beta values representing percentage differences in volumetric mammographic density per 1-SD increase in insulin genetic score

Associations of the insulin genetic score with MD are shown in Fig. 2, and a summary of individual SNP estimates is provided in Additional file 1: Table S9. Genetically predicted insulin levels were positively associated with both percent dense and absolute dense volume (Fig. 2) (beta [95% CI] per 1-SD increase in genetic score = 0.80 [0.00–1.59] and 0.93 [0.05–1.81], respectively), but not with the absolute nondense volume (beta [95% CI] per 1-SD increase in genetic score = 0.05 [− 0.78; 0.87]). There was no evidence of associations being driven by potential confounders (Additional file 4: Figure S2), except for genetically elevated insulin levels being weakly associated with a lower odds of previous benign breast disease. Given the positive association between benign breast disease and MD, analyses conditioning on this variable strengthened the effect estimates (Additional file 5: Figure S3).

## Discussion

In this mammography screening cohort, we found that insulin-treated T1D and T2D patients had higher MD levels as compared with age-matched individuals without diabetes and diabetes patients receiving other noninsulin glucose-lowering medication. Associations with the absolute dense volume were driven mainly by long-term insulin use and the long-acting insulin analog glargine, whereas no associations with treatment duration or insulin type were found for the absolute nondense volume. We further observed positive associations of genetically predicted insulin levels with percent dense and absolute dense volumes, but not with absolute nondense volume. The consistency in direction of association for insulin treatment and the insulin genetic score with the absolute dense volume strengthen the evidence of a potential causal effect of long-term increased insulin exposure on mammographic dense breast tissue.

Observational studies and randomized clinical trials are limited in their ability to investigate associations of long-term insulin exposure with relatively rare outcomes such as breast cancer. As a continuous intermediate trait, MD represents an attractive endpoint for identifying potential carcinogenic effects [40]. Because MD was routinely collected in all study participants regardless of screening outcome, this outcome is also not susceptible to ascertainment bias. To date, only one study assessed associations between exogenous insulin use and MD. This study [41] reported a suggestive association of insulin therapy with a higher prevalence of mixed/dense breast density patterns, but it included only 20 diabetes patients and could not examine associations with treatment duration or insulin glargine use, nor could it distinguish associations based on fibroglandular dense and adipose nondense tissues. Though previous observational data on breast cancer risk have been somewhat conflicting, recent findings [14] of a positive association between long-term insulin glargine use and breast cancer risk cohere with the results for the absolute dense volume reported herein. Altogether, these findings suggest that the carcinogenic potential of exogenous insulins might be greatest for insulin glargine, possibly because of its unique characteristics in terms of receptor affinity and pharmacokinetic profile with prolonged duration of action.

Because our study is observational in nature, we aimed to integrate evidence from different methodological approaches to assess the likelihood of a potential causal effect of long-term insulin exposure. First, we investigated the influence of exogenous insulin use in diabetes patients. To address confounding by indication, we assessed associations with insulin-treated T1D and T2D separately. Because T1D and T2D differ in pathophysiology and underlying risk factors (with T1D being an autoimmune disease, whereas insulin resistance, driven mainly by obesity, is the hallmark of T2D), consistent results for insulin-treated T1D and T2D are suggestive of an insulin therapy effect independent of underlying disease etiology. This analysis approach using nondiabetics as a comparator, however, does not rule out confounding by the indication itself (T1D or T2D). Hence, to address possible residual confounding, we also assessed MD differences by duration of insulin treatment and insulin glargine use in case-only analyses, and we performed additional analyses using non-insulin-treated diabetes patients as an active comparator. Although none of these observational assessments may be completely free of bias, sources and directions of bias in each of these are likely to be different. Therefore, consistency of direction of association across the different approaches can be interpreted as evidence for a potential causal association. To further investigate the likelihood of a long-term insulin effect, we also explored associations with an insulin genetic score [24] as an instrument for long-term exposure to elevated circulating insulin levels. Although effect sizes for this endogenous genetic proxy are not directly comparable to those observed for exogenous insulins, because of differences in measurement scale and magnitudes of anticipated physiological effects [13], the consistency in direction of associations observed for the absolute dense volume strengthen the evidence of a causal influence of long-term elevated insulin levels on mammographic dense tissue. On the other hand, direction of association for insulin treatment and the insulin genetic score were not consistent for the absolute nondense volume, arguing against a causal influence of insulin on adipose breast tissue.

To our knowledge, this is the first study investigating associations of long-term insulin exposure with MD combining data from observational and genetic analyses. Although absolute mammographic dense tissue is a well-known risk factor and intermediate phenotype of breast cancer, further studies investigating the effect of  long-term insulin exposure on breast cancer risk are warranted, ideally with MD measurement to address the extent to which breast cancer risk associations are mediated by MD. Because long-term exogenous insulin use in diabetes patients may have a greater impact on breast tissue than genetic predisposition to long-term elevations in endogenous insulin, it will also be relevant to assess potential differential associations by exposure type. Moreover, because insulin is the mainstay of treatment for T1D and uncontrolled T2D, and because diabetes patients tend to participate less in mammography screening programs [42, 43], increasing screening awareness and participation among insulin-treated patients with diabetes may be a first step in reducing insulin-associated adverse effects from a clinical perspective.

Some limitations of the present study are noteworthy. Historical information on insulin treatment duration was not complete, because the Prescribed Drug Register has had nationwide coverage since July 2005. This limited the analysis contrasting short- vs. long-term effects of insulin use, especially in insulin-treated T2D patients, where no assumption regarding treatment initiation could be made. Also, insulin glargine and treatment duration analyses were limited by small numbers of patients, which resulted in some uncertainty in effect estimates and low statistical power when analyses were restricted to diabetes patients only. We also cannot rule out misclassification of T1D and T2D patients in instances where diagnostic age cutoffs were used. T2D incidence below the age of 30 years was low in Sweden during the study period, and most diabetes patients diagnosed before 30 years of age are likely to be true T1D cases. Finally, our study was insufficiently powered to study effects of insulin analogs other than insulin glargine. Strengths of the current study are the screening-based setting, extensive information on potential confounders, and the use of a fully automated method for MD measurement that is not prone to subjective measurement error. The unique study design further allowed us to address the likelihood of a causal association by making relevant patient comparisons and by using a genetic score robustly associated with circulating insulin levels independent of other metabolic markers and confounding factors [24].

## Conclusions

Our study provides evidence of observational and genetic associations of long-term increased insulin exposure with the absolute dense volume. Apart from identifying a potential causal effect of long-term increased insulin exposure on mammographic dense breast tissue, these findings support efforts to improve screening awareness and participation among insulin-treated patients with diabetes.

## Additional files

Additional file 1: **Table S1.** Prescriptions of insulin and insulin analogs dispensed in the year prior to study entry in T1D and T2D patients. **Table S2.** Prescriptions of glucose-lowering medication dispensed in the year prior to study entry in T2D patients not receiving insulin therapy. **Table S3.** Single-nucleotide polymorphisms included in the insulin genetic score. **Table S4.** Descriptive characteristics of insulin-treated T2D patients and non-insulin-treated T2D patients. **Table S5.** Geometric means and percentage differences of volumetric mammographic density comparing glargine insulin users to non-glargine insulin users (case only analyes). **Table S6.** Geometric means and percentage differences of volumetric mammographic density comparing insulin-treated T2D patients to non-insulin treated T2D patients. **Table S7.** Geometric means and percentage differences of volumetric mammographic density comparing insulin-treated T1D and T2D patients to age-matched individuals without diabetes by insulin treatment duration. **Table S8.** Geometric means and percentage differences of volumetric mammographic density by treatment duration in insulin-treated T1D patients (case only analyses). **Table S9.** Associations of fasting insulin single nucleotide polymorphisms with volumetric mammographic density. (DOCX 88 kb)

Additional file 2: Supplementary methods: handing of missing covariate data. (DOCX 13 kb)

Additional file 3: **Figure S1.** Summary of percentage differences in volumetric mammography density observed by insulin therapy across the different observational analyses. Association of insulin therapy with volumetric mammographic density in different observational analyses with age and BMI adjusted estimates (*open circles)* and multivariable adjusted estimates (*closed circles*). Betas represent % differences in percent dense, absolute dense and absolute non-dense volume for differences in insulin exposure. (PDF 476 kb)

Additional file 4: **Figure S2.** Association of the insulin genetic score with potential confounders. Association of 18-SNP insulin genetic score with potential confounders in Karma sub-cohort of non-diabetic women with genotyping data. Associations were tested using linear regression and (multinomial) logistic regression, adjusting for age, six principal components and genotyping array. Betas represent differences in covariate level per 1-standard deviation increment in insulin genetic score. (PDF 225 kb)

Additional file 5: **Figure S3.** Association of the insulin genetic score with volumetric mammographic density measures after additional adjustment for benign breast disease. Association of 18-SNP insulin genetic score with volumetric mammographic density in Karma sub-cohort of non-diabetic women with genotyping data, overall and stratified by genotyping array and with additional adjustment for benign breast disease. Associations with volumetric mammographic density were tested by linear regression, adjusting for age, body mass index, menopausal status, six principal components and benign breast disease. Analyses in total sub-cohort were additionally adjusted for genotyping array. All volumetric mammographic density measures were log-transformed prior to analyses, with betas representing % differences in volumetric mammographic density per 1-standard deviation increment in insulin genetic score. (PDF 146 kb)

## Abbreviations

ATC: Anatomical Therapeutic Chemical Classification System
BMI: Body mass index
ICD: International Classification of Diseases
iCOGS: Illumina iSelect array
IGF-1: Insulin like-growth factor 1
Karma: KARolinska MAmmography Project for Risk Prediction of Breast Cancer
MD: Mammographic density
SNP: Single-nucleotide polymorphism
T1D: Type 1 diabetes
T2D: Type 2 diabetes
