# Supplementary material for: Long-term exposure to insulin and volumetric mammographic density: observational and genetic associations in the Karma study
Source: Breast Cancer Res. 2018 Aug 9;20:93. doi: 10.1186/s13058-018-1026-7 (PMC6085687; doi:10.1186/s13058-018-1026-7)
Supplement: Supplementary file 1 — Table S1. Prescriptions of insulin and insulin analogs dispensed in the year prior to study entry in T1D and T2D patients. Table S2. Prescriptions of glucose-lowering medication dispensed in the year prior to study entry in T2D patients not receiving insulin therapy. Table S3. Single-nucleotide polymorphisms included in the insulin genetic score. Table S4. Descriptive characteristics of insulin-treated T2D patients and non-insulin-treated T2D patients. Table S5. Geometric means and percentage differences of volumetric mammographic density comparing glargine insulin users to non-glargine insulin users (case only analyes). Table S6. Geometric means and percentage differences of volumetric mammographic density comparing insulin-treated T2D patients to non-insulin treated T2D patients. Table S7. Geometric means and percentage differences of volumetric mammographic density comparing insulin-treated T1D and T2D patients to age-matched individuals without diabetes by insulin treatment duration. Table S8. Geometric means and percentage differences of volumetric mammographic density by treatment duration in insulin-treated T1D patients (case only analyses). Table S9. Associations of fasting insulin single nucleotide polymorphisms with volumetric mammographic density. (DOCX 88 kb) [file 13058_2018_1026_MOESM1_ESM.docx]

**Supplementary Table 1.** Prescriptions of insulin and insulin analogs dispensed in the year prior to study entry in T1D and T2D patients.

|  |  |  | **N *** |  |
| --- | --- | --- | --- | --- |
| **Human insulin / insulin analogs** | **Type** | **ATC** | **T1D patients** | **T2D patients** |
| Human insulin | Fast-acting | A10AB01 | 4 | 3 |
| Insulin lispro | Fast-acting | A10AB04 | 55 | 34 |
| Insulin aspart | Fast-acting | A10AB05 | 61 | 99 |
| Insulin glulisine | Fast-acting | A10AB06 | 3 | 2 |
| Human insulin | Intermediate-acting | A10AC01 | 16 | 91 |
| Insulin lispro | Intermediate/long acting combined with fast acting | A10AD04 | 2 | 9 |
| Insulin apart | Intermediate/long acting combined with fast acting | A10AD05 | 2 | 45 |
| Insulin glargine | Long-acting | A10AE04 | 65 | 74 |
| Insulin detemir | Long-acting | A10AE05 | 23 | 31 |

Abbreviations: T1D = type 1 diabetes; T2D = type 2 diabetes; ATC = anatomical therapeutic chemical coding. * The number of prescriptions is larger than the number of insulin-treated individuals due to combination therapies and possible switches in therapy regimen in the year prior to study entry.

**Supplementary Table 2.** Prescriptions of glucose-lowering medication dispensed in the year prior to study entry in T2D patients not receiving insulin therapy.

|  |  |  | **N *** |
| --- | --- | --- | --- |
| **Glucose-lowering medication** | **Class** | **ATC** | **T2D patients** |
| Metformin | Metformin | A10BA02 | 371 |
| [Glibenclamide](https://www.whocc.no/atc_ddd_index/?code=A10BB01&showdescription=yes) | Sulfonylureas | A10BB01 | 7 |
| [Glipizide](https://www.whocc.no/atc_ddd_index/?code=A10BB07&showdescription=yes) | Sulfonylureas | A10BB07 | 6 |
| [Glimepiride](https://www.whocc.no/atc_ddd_index/?code=A10BB12&showdescription=yes) | Sulfonylureas | A10BB12 | 5 |
| Metformin and [pioglitazone](https://www.whocc.no/atc_ddd_index/?code=A10BG03&showdescription=yes) | Combination of blood glucose lowering drugs | A10BD05 | 1 |
| [Metformin and sitagliptin](https://www.whocc.no/atc_ddd_index/?code=A10BD07&showdescription=yes) | Combination of blood glucose lowering drugs | A10BD07 | 1 |
| [Pioglitazone](https://www.whocc.no/atc_ddd_index/?code=A10BG03&showdescription=yes) | [Thiazolidinediones](https://www.whocc.no/atc_ddd_index/?code=A10BG) | A10BG03 | 2 |
| [Sitagliptin](https://www.whocc.no/atc_ddd_index/?code=A10BH01&showdescription=yes) | [Dipeptidyl peptidase 4 (DPP-4) inhibitors](https://www.whocc.no/atc_ddd_index/?code=A10BH) | A10BH01 | 1 |
| [Repaglinide](https://www.whocc.no/atc_ddd_index/?code=A10BX02&showdescription=yes) | Other blood glucose-lowering drugs | A10BX02 | 11 |
| Liraglutide | Other blood glucose-lowering drugs | A10BX07 | 2 |

Abbreviations: T2D = type 2 diabetes; ATC = anatomical therapeutic chemical coding. * The number of prescriptions is larger than the number of individuals on glucose-lowering therapy due to combination therapies and possible switches in therapy regimen in the year prior to study entry.

**Supplementary Table 3.** Single nucleotide polymorphisms included in the insulin genetic score.

|  |  |  |  |  | **iCOGS sub-cohort**  **(N = 3,909)** | | **OncoArray sub-cohort**  **(N = 5,528)** | |
| --- | --- | --- | --- | --- | --- | --- | --- | --- |
| **CHR** | **BP** | **SNP** | **A1** | **A2** | **MAF** | **INFO** | **MAF** | **INFO** |
| 1 | 219722104 | rs4846565 | G | A | 0.34 | 1.00 | 0.34 | 1.00 |
| 2 | 27741237 | rs780094 | C | T | 0.35 | 1.00 | 0.35 | 1.00 |
| 2 | 135755629 | rs1530559 | G | A | 0.35 | 0.75 | 0.37 | 0.84 |
| 2 | 165513091 | rs10195252 | T | C | 0.44 | 1.00 | 0.44 | 1.00 |
| 2 | 227099180 | rs2943645 | T | C | 0.38 | 0.98 | 0.38 | 1.00 |
| 3 | 12390484 | rs17036328 | T | C | 0.14 | 0.97 | 0.15 | 1.00 |
| 4 | 89741269 | rs3822072 | G | A | 0.44 | 0.94 | 0.45 | 0.99 |
| 4 | 106071064 | rs974801 | A | G | 0.40 | 1.00 | 0.41 | 1.00 |
| 4 | 157734675 | rs6822892 | A | G | 0.32 | 0.93 | 0.31 | 0.97 |
| 5 | 53272664 | rs4865796 | A | G | 0.31 | 0.96 | 0.31 | 0.99 |
| 5 | 55806751 | rs459193 | G | A | 0.28 | 0.70 | 0.26 | 1.00 |
| 6 | 34764922 | rs6912327 | T | C | 0.17 | 0.99 | 0.18 | 1.00 |
| 6 | 127452935 | rs2745353 | C | T | 0.44 | 0.90 | 0.46 | 1.00 |
| 7 | 75176196 | rs1167800 | A | G | 0.43 | 0.99 | 0.42 | 0.98 |
| 8 | 9185146 | rs2126259 | C | T | 0.11 | 0.99 | 0.11 | 1.00 |
| 10 | 114758349 | rs7903146 | C | T | 0.25 | 1.00 | 0.26 | 1.00 |
| 12 | 102898446 | rs860598 | A | G | 0.17 | 1.00 | 0.17 | 0.99 |
| 19 | 33899065 | rs731839 | A | G | 0.34 | 0.86 | 0.33 | 1.00 |

Single nucleotide polymorphisms associated with fasting insulin levels. Abbreviations: CHR = chromosome; BP = base pair position; SNP = single nucleotide polymorphism; A1 = major allele; A2 = minor allele; MAF = minor allele frequency; INFO = Information score for imputation; 1.00 corresponds to a genotyped variant. Average INFO score for all SNPs = 0.94 (iCOGS) and 0.99 (OncoArray).

**Supplementary Table 4.** Descriptive characteristics of insulin-treated T2D patients and non-insulin treated T2D patients.

| **Characteristic** | **Insulin-treated T2D**  **(N = 112)** | **Non-insulin treated T2D**  **(N = 407)** | ***P* value** |
| --- | --- | --- | --- |
| Age (years), mean (SD) | 62.0 (8.7) | 63.4 (7.4) | 0.07 |
| BMI (kg/m^2^), mean (SD) | 27.6 (5.6) | 29.7 (5.3) | < 0.001 |
| Education level, % (N) |  |  | 0.40 |
| Compulsory | 28.4 (29) | 31.6 (121) |  |
| Gymnasium | 27.5 (28) | 31.6 (121) |  |
| University | 44.1 (45) | 36.8 (141) |  |
| *Missing* | *8.9 (10)* | *5.9 (24)* |  |
| Age at menarche (years), mean (SD) | 12.9 (1.5) | 13.1 (1.6) | 0.30 |
| Parity, % (N) |  |  | 0.57 |
| 0 | 16.4 (18) | 11.5 (46) |  |
| 1 | 14.5 (16) | 16.7 (67) |  |
| 2 | 45.5 (50) | 46.4 (186) |  |
| ≥ 3 | 23.6 (26) | 25.4 (102) |  |
| *Missing* | *1.8 (2)* | *1.5 (6)* |  |
| Age at first birth (years), mean (SD) | 24.4 (4.9) | 24.7 (4.6) | 0.58 |
| *Missing* | *5.4 (5)* | *9.9 (35)* |  |
| Menopausal status, % (N) |  |  | 0.07 |
| Premenopausal | 17.9 (20) | 11.3 (46) |  |
| Postmenopausal | 82.1 (92) | 88.7 (361) |  |
| OC use (ever), % (N) | 67.3 (74) | 70.3 (279) | 0.54 |
| *Missing* | *1.8 (2)* | *2.5 (10)* |  |
| HRT, % (N) |  |  | 0.80 |
| Never | 72.5 (74) | 69.3 (257) |  |
| Former | 24.5 (25) | 27.8 (103) |  |
| Current | 2.9 (3) | 3.0 (11) |  |
| *Missing* | *8.9 (10)* | *8.8 (36)* |  |
| Alcohol intake, % (N) |  |  | 0.35 |
| None | 40.4 (44) | 39.7 (159) |  |
| 1-25 g/week | 15.6 (17) | 22.7 (91) |  |
| 25-50 g/week | 18.3 (20) | 17.5 (70) |  |
| >50 g/week | 25.7 (28) | 20.2 (81) |  |
| *Missing* | *2.7 (3)* | *1.5 (6)* |  |

**Supplementary Table 4.** *continued*

| **Characteristic** | **Insulin-treated T2D**  **(N = 112)** | **Non-insulin treated T2D**  **(N = 407)** | ***P* value** |
| --- | --- | --- | --- |
| Physical activitiy, % (N) |  |  | 0.74 |
| < 40 MET hrs/day | 51.4 (54) | 49.4 (193) |  |
| 40-45 MET hrs/day | 32.4 (34) | 37.1 (145) |  |
| 45-50 MET hrs/day | 11.4 (12) | 8.7 (34) |  |
| >50 MET hrs/day | 4.8 (5) | 4.9 (19) |  |
| *Missing* | *6.3 (7)* | *3.9 (16)* |  |
| Smoking status, % (N) |  |  | 0.97 |
| Never | 40.5 (45) | 39.2 (159) |  |
| Former | 43.2 (48) | 44.3 (180) |  |
| Current | 16.2 (18) | 16.5 (67) |  |
| *Missing* | *0.9 (1)* | *0.2 (1)* |  |
| Statin therapy (current), % (N) | 54.5 (61) | 61.9 (252) | 0.15 |
| Low dose aspirin (current), % (N) | 25.0 (28) | 30.2 (123) | 0.28 |
| Charlson comorbidity index, % (N) |  |  | < 0.001 |
| 0 | 75.9 (85) | 81.6 (332) |  |
| 1 | 9.8 (11) | 15.0 (61) |  |
| ≥ 2 | 14.3 (16) | 3.4 (14) |  |
| Benign breast disease, % (N) |  |  | 0.01 |
| No | 69.7 (76) | 80.8 (325) |  |
| Yes | 30.3 (33) | 19.2 (77) |  |
| *Missing* | *2.7 (3)* | *1.2 (5)* |  |
| Family history of breast cancer, % (N) |  |  | 0.41 |
| No | 83.0 (88) | 86.2 (325) |  |
| Yes | 17.0 (18) | 13.8 (52) |  |
| *Missing* | *5.4 (6)* | *7.4 (30)* |  |
| Age at diagnosis (years), mean (SD) | 53.7 (8.1) | 58.6 (7.7) | < 0.001 |
| Diabetes duration (years), mean (SD) | 8.3 (5.6) | 4.8 (4.2) | < 0.001 |
| Insulin therapy, % (N) |  |  |  |
| Glargine insulin | 45.5 (51) | - |  |
| Non-glargine insulin | 54.5 (61) | - |  |
| Glucose-lowering therapy, % (N) |  |  |  |
| Metformin | *-* | 91.2 (371) |  |
| Other | - | 8.8 (36) |  |

Study population for active comparator analysis comparing T2D patients treated with insulin only to T2D patients receiving other non-insulin glucose-lowering medication. All participants were free of cancer at the baseline screening visit. Characteristics were compared using T-tests for continuous data and Chi-square tests for categorical variables. Abbreviations: T1D = type 1 diabetes; T2D = type 2 diabetes; BMI = body mass index; OC = oral contraceptive; HRT = hormone replacement therapy; MET = metabolic equivalent of activity level.

**Supplementary Table 5.** Geometric means and percentage differences of volumetric mammographic density comparing glargine insulin users to non-glargine insulin users (case only analyses).

|  |  | **Geometric mean (95% CI)** | | | | | |
| --- | --- | --- | --- | --- | --- | --- | --- |
|  |  | **Percent dense volume** | | **Absolute dense volume** | | **Absolute non-dense volume** | |
|  | **N** | **Age and BMI adjusted** | **Multivariable**  **adjusted** | **Age and BMI**  **adjusted** | **Multivariable**  **Adjusted** | **Age and BMI**  **adjusted** | **Multivariable**  **adjusted** |
| **Insulin-treated T1D** |  |  |  |  |  |  |  |
| T1D - non-glargine insulin | 52 | 10.9 (9.7; 12.1) | 11.0 (9.8- 12.4) | 60.4 (53.6; 68.0) | 60.8 (53.7; 68.9) | 485.8 (435.8; 541.7) | 480.8 (430.0; 537.8) |
| T1D - glargine insulin | 65 | 11.1 (10.0; 12.2) | 10.9 (9.8- 12.1) | 71.6 (64.4; 79.6) | 71.2 (63.8; 79.5) | 562.8 (510.7; 620.1) | 567.5 (514.1; 626.4) |
|  |  | 0.81 | 0.91 | 0.04 | 0.07 | 0.05 | 0.04 |
| **Insulin-treated T2D** |  |  |  |  |  |  |  |
| T2D - non-glargine insulin | 61 | 6.5 (5.8; 7.2) | 6.4 (5.7; 7.2) | 58.4 (52.5; 65.0) | 57.4 (51.1; 64.4) | 835.6 (747.4; 934.2) | 826.6 (735.8; 928.6) |
| T2D - glargine insulin | 51 | 7.4 (6.6; 8.4) | 7.5 (6.6; 8.5) | 64.3 (57.2; 72.4) | 65.7 (57.9; 74.7) | 793.6 (702.2; 896.9) | 804.0 (707.1; 914.0) |
|  |  | 0.11 | 0.11 | 0.24 | 0.14 | 0.54 | 0.76 |
|  |  | **Percentage difference (95% CI)** | | | | | |
|  |  | **Percent dense volume** | | **Absolute dense volume** | | **Absolute non-dense volume** | |
|  | **N** | **Age and BMI adjusted** | **Multivariable**  **adjusted** | **Age and BMI**  **adjusted** | **Multivariable**  **Adjusted** | **Age and BMI**  **adjusted** | **Multivariable**  **adjusted** |
| **Insulin-treated T1D** |  |  |  |  |  |  |  |
| T1D - non-glargine insulin | 52 | REF | REF | REF | REF | REF | REF |
| T1D - glargine insulin | 65 | 1.8 (-12.3; 18.1) | -0.9 (-16.2; 17.1) | 18.6 (0.9; 39.3) | 17.1 (-1.7; 39.6) | 15.8 (0.0; 34.2) | 18.0 (0.8; 38.1) |
| **Insulin-treated T2D** |  |  |  |  |  |  |  |
| T2D - non-glargine insulin | 61 | REF | REF | REF | REF | REF | REF |
| T2D - glargine insulin | 51 | 14.4 (-3.0; 34.9) | 16.0 (-3.4; 39.4) | 10.2 (-6.3; 29.5) | 14.6 (-4.3; 37.1) | -5.0 (-19.7; 12.3) | -2.7 (-18.9; 16.6) |

Abbreviations: T1D = type 1 diabetes; T2D = type 2 diabetes; BMI = body mass index; REF = reference group. Multivariable adjusted model: model adjusted for age (years), body mass index (kg/m^2^), education level (compulsory, gymnasium, university), age at menarche (years), parity and age at first birth (nulliparous, parous / age at first birth < 25 years, parous / age at first birth 25-30 years, parous /age at first birth > 30 years), oral contraceptives (never, ever), menopausal status (premenopausal, postmenopausal), hormone replacement therapy (never, former, current), alcohol intake (none, 1-25 g/week, 25-50 g/week, > 50 g/week), physical activity (< 40 MET hrs/day, 40-45 MET hrs/day, 45-50 MET hrs/day, > 50 MET hrs/day), smoking status (never, former, current), statins (no, yes), low-dose aspirin (no, yes), Charlson comorbidity index (0,1,≥2), benign breast disease (no, yes), family history of breast cancer (no, yes) and metformin therapy (no, yes).

**Supplementary Table 6.** Geometric means and percentage differences of volumetric mammographic density comparing insulin-treated T2D patients to non-insulin treated T2D patients.

|  |  | **Geometric mean (95% CI)** | | | | | |
| --- | --- | --- | --- | --- | --- | --- | --- |
|  |  | **Percent dense volume (%)** | | **Absolute dense volume (cm^3^)** | | **Absolute non-dense volume (cm^3^)** | |
|  | **N** | **Age and BMI adjusted** | **Multivariable**  **adjusted** | **Age and BMI**  **adjusted** | **Multivariable**  **adjusted** | **Age and BMI**  **adjusted** | **Multivariable**  **Adjusted** |
| **Insulin-treated T2D** |  |  |  |  |  |  |  |
| Non-insulin-treated T2D | 407 | 5.1 (4.9; 5.2) | 5.1 (4.9; 5.2) | 57.5 (55.4; 59.8) | 57.5 (55.3; 59.8) | 1075.6 (1039.5; 1112.9) | 1075.2 (1038.1; 1113.6) |
| T2D - insulin any | 112 | 6.4 (5.9; 6.8) | 6.4 (5.9; 6.9) | 62.6 (58.1; 67.3) | 62.8 (58.0; 67.9) | 909.7 (851.8; 971.4) | 910.8 (848.3; 977.9) |
| *P* value |  | < 0.001 | < 0.001 | 0.001 | 0.06 | < 0.001 | < 0.001 |
| Non-insulin-treated T2D | 407 | 5.1 (4.9; 5.2) | 5.0 (4.9; 5.2) | 57.5 (55.4; 59.7) | 57.5 (55.3; 59.7) | 1075.8 (1039.7; 1113.1) | 1075.3 (1038.2; 1113.7) |
| T2D - non-glargine insulin | 61 | 5.9 (5.4; 6.5) | 5.9 (5.3; 6.5) | 59.3 (53.8; 65.4) | 59.3 (53.5; 65.7) | 939.0 (860.0; 1025.2) | 940.9 (857.0; 1032.9) |
| T2D - glargine insulin | 51 | 7.0 (6.3; 7.8) | 7.0 (6.3; 7.9) | 66.9 (60.0; 74.6) | 67.3 (60.1; 75.3) | 874.4 (792.7; 964.4) | 875.8 (790.7; 969.9) |
| *P* value |  | < 0.001 | < 0.001 | 0.04 | 0.04 | < 0.001 | < 0.001 |
|  |  | **Percentage difference (95% CI)** | | | | | |
|  |  | **Percent dense volume** | | **Absolute dense volume** | | **Absolute non-dense volume** | |
|  | **N** | **Age and BMI adjusted** | **Multivariable**  **adjusted** | **Age and BMI adjusted** | **Multivariable**  **adjusted** | **Age and BMI**  **adjusted** | **Multivariable**  **Adjusted** |
| **Insulin-treated T2D** |  |  |  |  |  |  |  |
| Non-insulin treated T2D | 407 | REF | REF | REF | REF | REF | REF |
| T2D - insulin any | 112 | 26.0 (16.2; 36.6) | 26.5 (15.9; 38.1) | 8.8 (0.1; 18.2) | 9.2 (-0.2; 19.6) | -15.4 (-21.5; -8.9) | -15.3 (-22.0; -8.0) |
| Non-insulin treated T2D | 407 | REF | REF | REF | REF | REF | REF |
| T2D - non-glargine insulin | 61 | 16.7 (5.4; 29.2) | 16.7 (4.7; 30.0) | 3.1 (-7.2; 14.6) | 3.1 (-7.8; 15.4) | -12.7 (-20.6; -4.1) | -12.5 (-21.0; -3.1) |
| T2D - glargine insulin | 51 | 38.7 (23.9; 55.3) | 39.6 (24.2; 57.0) | 16.3 (3.5; 30.7) | 17.1 (3.7; 32.2) | -18.7 (-26.8; -9.8) | -18.6 (-27.0; -9.1) |

Abbreviations: T2D = type 2 diabetes; BMI = body mass index; REF = reference group. Multivariable adjusted model: model adjusted for age (years), body mass index (kg/m^2^), education level (compulsory, gymnasium, university), age at menarche (years), parity and age at first birth (nulliparous, parous / age at first birth < 25 years, parous / age at first birth 25-30 years, parous /age at first birth > 30 years), menopausal status, oral contraceptives (never, ever), menopausal status (premenopausal, postmenopausal), hormone replacement therapy (never, former, current), alcohol intake (none, 1-25 g/week, 25-50 g/week, > 50 g/week), physical activity (< 40 MET hrs/day, 40-45 MET hrs/day, 45-50 MET hrs/day, > 50 MET hrs/day), smoking status (never, former, current), statins (no, yes), low-dose aspirin (no, yes), Charlson comorbidity index (0,1,≥2), benign breast disease (no, yes), family history of breast cancer (no, yes) and disease duration (years).

**Supplementary Table 7.** Geometric means and percentage differences of volumetric mammographic density comparing insulin-treated T1D and T2D patients to age-matched individuals without diabetes by insulin treatment duration.

|  |  | **Geometric mean (95% CI)** | | | | | |
| --- | --- | --- | --- | --- | --- | --- | --- |
|  |  | **Percent dense volume (%)** | | **Absolute dense volume (cm^3^)** | | **Absolute non-dense volume (cm^3^)** | |
|  | **N** | **Age and BMI adjusted** | **Multivariable**  **adjusted** | **Age and BMI**  **Adjusted** | **Multivariable**  **adjusted** | **Age and BMI**  **adjusted** | **Multivariable**  **adjusted** |
| **Insulin-treated T1D** | |  |  |  |  |  |  |
| Non-diabetics | 610 | 8.8 (8.5; 9.0) | 8.8 (8.5; 9.1) | 59.6 (57.3; 61.9) | 59.8 (57.6; 62.2) | 610.8 (590.4; 631.8) | 611.6 (590.8; 633.2) |
| T1D - insulin 10-27 years | 45 | 10.6 (9.3; 11.9) | 10.3 (9.1; 11.6) | 61.6 (53.4; 70.9) | 57.2 (49.5; 66.2) | 483.8 (425.5; 550.2) | 489.6 (429.4; 558.4) |
| T1D - insulin 28-36 years | 40 | 11.3 (10.0; 12.8) | 11.1 (9.8; 12.7) | 67.2 (58.0; 77.9) | 65.9 (56.4; 77.1) | 512.9 (449.4; 585.4) | 514.9 (447.3; 592.7) |
| T1D - insulin > 36 years | 37 | 12.1 (10.6; 13.8) | 12.1 (10.4; 14.0) | 69.6 (59.6; 81.1) | 72.0 (60.6; 85.6) | 532.8 (462.8; 613.2) | 510.9 (436.8; 597.5) |
| **Insulin-treated T2D** | |  |  |  |  |  |  |
| Non-diabetics | 1161 | 6.5 (6.4; 6.7) | 6.5 (6.3; 6.7) | 52.7 (51.4; 54.2) | 52.8 (51.4; 54.3) | 747.5 (729.0; 766.5) | 752.4 (733.5; 771.8) |
| T2D - insulin < 5 years | 83 | 7.5 (6.7; 8.3) | 7.7 (6.9; 8.6) | 55.6 (49.7; 62.2) | 54.9 (49.0; 61.5) | 670.5 (603.0; 745.5) | 650.9 (584.4; 725.0) |
| T2D - insulin ≥ 5 years | 154 | 7.3 (6.8; 7.9) | 7.5 (7.0; 8.2) | 58.3 (53.8; 63.0) | 58.0 (53.3; 63.0) | 726.5 (674.2; 782.8) | 702.9 (649.0; 761.1) |
|  |  | **Percentage difference (95% CI)** | | | | | |
|  |  | **Percent dense volume** | | **Absolute dense volume** | | **Absolute non-dense volume** | |
|  | **N** | **Age and BMI adjusted** | **Multivariable**  **adjusted** | **Age and BMI**  **Adjusted** | **Multivariable**  **adjusted** | **Age and BMI**  **adjusted** | **Multivariable**  **adjusted** |
| **Insulin-treated T1D** |  |  |  |  |  |  |  |
| Non-diabetics | 610 | REF | REF | REF | REF | REF | REF |
| T1D - insulin 10-27 years | 45 | 20.6 (6.3; 36.8) | 17.0 (2.9; 33.2) | -2.1 (-15.6; 13.5) | -4.4 (-17.9; 11.3) | -20.8 (-30.7; -9.5) | -19.9 (-30.2; -8.2) |
| T1D - insulin 28-36 years | 40 | 29.2 (13.6; 47.0) | 26.9 (10.5; 45.8) | 11.9 (-3.8; 30.2) | 10.2 (-6.4; 29.8) | -16.0 (-26.8; -3.7) | -15.8 (-27.4; -2.4) |
| T1D - insulin > 36 years | 37 | 37.8 (20.2; 58.0) | 37.9 (18.3; 60.8) | 25.4 (6.8; 47.2) | 20.4 (0.4; 44.2) | -12.8 (-24.5; 0.8) | -16.5 (-29.1; -1.6) |
| **Insulin-treated T2D** |  |  |  |  |  |  |  |
| Non-diabetics | 1161 | REF | REF | REF | REF | REF | REF |
| T2D - insulin < 5 years | 83 | 14.6 (2.0; 28.7) | 18.4 (5.2; 33.3) | 4.0 (-7.7; 17.2) | 3.9 (-8.0; 17.4) | -10.3 (-20.0; 0.6) | -13.5 (-23.0; -2.8) |
| T2D - insulin ≥ 5 years | 154 | 12.3 (3.2; 22.2) | 16.0 (5.9; 27.1) | 10.3 (1.1; 20.4) | 9.7 (-0.1; 20.5) | -2.8 (-10.6; 5.6) | -6.6 (-14.6; 2.2) |

Abbreviations: T1D = type 1 diabetes; T2D = type 2 diabetes; BMI = body mass index; REF = reference group. Multivariable adjusted model: model adjusted for age (years), body mass index (kg/m^2^), education level (compulsory, gymnasium, university), age at menarche (years), parity and age at first birth (nulliparous, parous / age at first birth < 25 years, parous / age at first birth 25-30 years, parous /age at first birth > 30 years), oral contraceptives (never, ever), menopausal status (premenopausal, postmenopausal), hormone replacement therapy (never, former, current), alcohol intake (none, 1-25 g/week, 25-50 g/week, > 50 g/week), physical activity (< 40 MET hrs/day, 40-45 MET hrs/day, 45-50 MET hrs/day, > 50 MET hrs/day), smoking status (never, former, current), statins (no, yes), low-dose aspirin (no, yes), Charlson comorbidity index (0,1,≥2), benign breast disease (no, yes), family history of breast cancer (no, yes) and metformin therapy (no, yes).

**Supplementary Table 8.** Geometric means and percentage differences of volumetric mammographic density by treatment duration in insulin-treated T1D patients (case only analyses).

|  |  | **Geometric mean (95% CI)** | | | | | |
| --- | --- | --- | --- | --- | --- | --- | --- |
|  |  | **Percent dense volume (%)** | | **Absolute dense volume (cm^3^)** | | **Absolute non-dense volume (cm^3^)** | |
|  | **N** | **Age and BMI adjusted** | **Multivariable**  **adjusted** | **Age and BMI**  **Adjusted** | **Multivariable**  **adjusted** | **Age and BMI**  **adjusted** | **Multivariable**  **Adjusted** |
| **Insulin-treated T1D** |  |  |  |  |  |  |  |
| T1D - insulin 10-27 years | 45 | 10.6 (9.2; 12.1) | 10.4 (8.9; 12.1) | 58.3 (50.0; 68.0) | 60.1 (50.9; 70.8) | 481.1 (419.8; 551.5) | 504.0 (435.2; 583.7) |
| T1D - insulin 28-36 years | 40 | 11.0 (9.7; 12.5) | 11.0 (9.6; 12.6) | 67.9 (59.1; 78.1) | 70.3 (60.7; 81.4) | 537.7 (475.2; 608.5) | 557.1 (488.6; 635.2) |
| T1D - insulin > 36 years | 37 | 10.8 (9.2; 12.6) | 11.0 (9.1; 13.1) | 76.3 (63.8; 91.3) | 70.9 (58.3; 86.3) | 623.3 (531.4; 731.1) | 567.0 (476.2; 675.1) |
| *P* trend |  | 0.84 | 0.68 | 0.04 | 0.23 | 0.04 | 0.34 |
|  |  | **Percentage difference (95% CI)** | | | | | |
|  |  | **Percent dense volume** | | **Absolute dense volume** | | **Absolute non-dense volume** | |
|  | **N** | **Age and BMI adjusted** | **Multivariable**  **adjusted** | **Age and BMI adjusted** | **Multivariable**  **adjusted** | **Age and BMI adjusted** | **Multivariable**  **Adjusted** |
| **Insulin-treated T1D** |  |  |  |  |  |  |  |
| T1D - insulin 10-27 years | 45 | REF | REF | REF | REF | REF | REF |
| T1D - insulin 28-36 years | 40 | 4.2 (-13.3; 25.2) | 5.3 (-14.3; 29.4) | 16.5 (-5.0; 42.9) | 17.0 (-6.5; 46.3) | 11.8 (-6.8; 34.0) | 10.5 (-9.4; 34.8) |
| T1D - insulin > 36 years | 37 | 1.8 (-20.2; 29.8) | 5.2 (-20.4; 38.9) | 30.9 (-0.1; 71.4) | 18.1 (-12.6; 59.5) | 29.5 (1.9; 64.8) | 12.5 (-14.0; 47.1) |

Abbreviations: T1D = type 1 diabetes; BMI = body mass index; REF = reference group. Multivariable adjusted model: model adjusted for age (years), body mass index (kg/m^2^), education level (compulsory, gymnasium, university), age at menarche (years), parity and age at first birth (nulliparous, parous / age at first birth < 25 years, parous / age at first birth 25-30 years, parous /age at first birth > 30 years), oral contraceptives (never, ever), menopausal status (premenopausal, postmenopausal), hormone replacement therapy (never, former, current), alcohol intake (none, 1-25 g/week, 25-50 g/week, > 50 g/week), physical activity (< 40 MET hrs/day, 40-45 MET hrs/day, 45-50 MET hrs/day, > 50 MET hrs/day), smoking status (never, former, current), statins (no, yes), low-dose aspirin (no, yes), Charlson comorbidity index (0,1,≥2), benign breast disease (no, yes), family history of breast cancer (no, yes) and metformin therapy (no, yes).

**Supplementary Table 9.** Associations of fasting insulin single nucleotide polymorphisms with volumetric mammographic density.

|  |  |  |  |  |  |  | **Insulin** | **Percent dense volume** | | | **Absolute dense volume** | | | **Absolute non-dense volume** | | |
| --- | --- | --- | --- | --- | --- | --- | --- | --- | --- | --- | --- | --- | --- | --- | --- | --- |
| **CHR** | **BP** | **SNP** | **Locus** | **Effect allele** | **Non-effect allele** | **EAF** | **beta *** | **beta** | **se** | ***P* value** | **beta** | **se** | ***P* value** | **beta** | **se** | ***P* value** |
| 1 | 219722104 | rs4846565 | *LYPLAL1* | G | A | 0.66 | 0.015 | 0.017 | 0.012 | 0.15 | 0.027 | 0.014 | 0.06 | 0.006 | 0.010 | 0.55 |
| 2 | 27741237 | rs780094 | *GCKR* | C | T | 0.65 | 0.022 | 0.015 | 0.012 | 0.21 | -0.001 | 0.014 | 0.93 | -0.015 | 0.010 | 0.14 |
| 2 | 135755629 | rs1530559 | *YSK4* | A | G | 0.64 | 0.013 | 0.006 | 0.013 | 0.62 | 0.015 | 0.016 | 0.35 | 0.006 | 0.011 | 0.62 |
| 2 | 165513091 | rs10195252 | *GRB14* | T | C | 0.56 | 0.018 | -0.010 | 0.011 | 0.38 | -0.001 | 0.014 | 0.94 | 0.008 | 0.010 | 0.41 |
| 2 | 227099180 | rs2943645 | *IRS1* | T | C | 0.62 | 0.016 | -0.028 | 0.011 | 0.01 | -0.020 | 0.014 | 0.15 | 0.012 | 0.010 | 0.21 |
| 3 | 12390484 | rs17036328 | *PPARG* | T | C | 0.86 | 0.014 | 0.046 | 0.016 | 3.95x10^-3^ | -0.001 | 0.019 | 0.96 | -0.042 | 0.014 | 2.31x10^-3^ |
| 4 | 89741269 | rs3822072 | *FAM13A1* | A | G | 0.45 | 0.010 | 0.000 | 0.011 | 0.98 | -0.001 | 0.014 | 0.96 | -0.002 | 0.010 | 0.85 |
| 4 | 106071064 | rs974801 | *TET2* | G | A | 0.41 | 0.016 | 0.034 | 0.011 | 2.11x10^-3^ | 0.032 | 0.014 | 0.02 | -0.007 | 0.010 | 0.49 |
| 4 | 157734675 | rs6822892 | *PDGFC* | A | G | 0.69 | 0.010 | 0.000 | 0.012 | 0.98 | 0.038 | 0.015 | 0.01 | 0.028 | 0.010 | 0.01 |
| 5 | 53272664 | rs4865796 | *ARL15* | A | G | 0.69 | 0.016 | 0.011 | 0.012 | 0.37 | 0.016 | 0.015 | 0.27 | 0.000 | 0.010 | 0.98 |
| 5 | 55806751 | rs459193 | *ANKRD55* | G | A | 0.74 | 0.019 | 0.013 | 0.013 | 0.36 | 0.003 | 0.016 | 0.86 | -0.010 | 0.012 | 0.39 |
| 6 | 34764922 | rs6912327 | *C6orf107* | T | C | 0.82 | 0.015 | -0.018 | 0.014 | 0.22 | -0.009 | 0.018 | 0.61 | 0.009 | 0.013 | 0.48 |
| 6 | 127452935 | rs2745353 | *RSPO3* | T | C | 0.55 | 0.015 | -0.008 | 0.011 | 0.46 | 0.022 | 0.014 | 0.10 | 0.025 | 0.010 | 0.01 |
| 7 | 75176196 | rs1167800 | *HIP1* | A | G | 0.57 | 0.015 | 0.026 | 0.011 | 0.02 | 0.017 | 0.014 | 0.22 | -0.012 | 0.010 | 0.20 |
| 8 | 9185146 | rs2126259 | *PPP1R3B* | T | C | 0.11 | 0.031 | -0.014 | 0.018 | 0.43 | -0.023 | 0.022 | 0.28 | -0.003 | 0.015 | 0.84 |
| 10 | 114758349 | rs7903146 | *TCF7L2* | C | T | 0.74 | 0.022 | 0.011 | 0.012 | 0.38 | -0.004 | 0.015 | 0.81 | 0.013 | 0.011 | 0.25 |
| 12 | 102898446 | rs860598 | *IGF1* | A | G | 0.83 | 0.012 | -0.013 | 0.015 | 0.38 | -0.005 | 0.018 | 0.79 | 0.009 | 0.013 | 0.47 |
| 19 | 33899065 | rs731839 | *PEPD* | G | A | 0.33 | 0.017 | 0.004 | 0.012 | 0.71 | 0.019 | 0.015 | 0.21 | 0.010 | 0.010 | 0.32 |

Association of 18 fasting insulin SNPs and volumetric mammographic density measures in Karma sub-cohort of non-diabetic women with genotyping data, from pooled analyses of Oncoarray and iCOGS samples. All betas and P values as derived from fixed effects meta-analyses. Abbreviations: CHR = chromosome; BP = base pair position; SNP = single nucleotide polymorphism; A1 = effect allele for fasting insulin levels; A2 = non-effect allele for fasting insulin levels; EAF = affect allele frequency in study sample (N = 9437). All volumetric mammographic density measures were log-transformed prior to analyses, with betas presenting differences in log transformed volumetric mammographic density per effect allele increase. * Literature-based betas representing differences in log fasting insulin levels per risk allele increase.
