# Supplementary material for: Long-term exposure to insulin and volumetric mammographic density: observational and genetic associations in the Karma study
Source: Breast Cancer Res. 2018 Aug 9;20:93. doi: 10.1186/s13058-018-1026-7 (PMC6085687; doi:10.1186/s13058-018-1026-7)
Supplement: Supplementary file 2 — Supplementary methods: handing of missing covariate data. (DOCX 13 kb) [file 13058_2018_1026_MOESM2_ESM.docx]

**Supplementary methods**

***Handing of missing covariate data***

All study participants had complete data on age, body mass index and menopausal status, drug prescriptions and comorbid conditions at study entry. Some participants had missing values on other covariates (see **Table 1** for a summary of missing values per participant characteristic). The number of participants with missing values on at least 1 covariate was higher in the sub-cohort for genetic analysis than in the matched cohort for insulin-treated T1D and T2D (30.7% vs. 21.7%) analyses. This is because the Karma sub-cohort for genetic analysis included a larger number of women who participated around cohort conception, a time at which the baseline questionnaire was not yet finalized. Because missing values were likely to be missing at random, missing values were imputed using multivariate multiple imputation with chained equations and 10 imputed datasets were generated (using “mi impute” in Stata). Imputation models were constructed for each individual exposure and included the exposure (insulin-treated T1D, insulin-treated T2D, insulin genetic score), outcome (mammographic density entered as percent dense volume), participants’ age and all other characteristics (body mass index, education level, age at menarche, parity and age at first birth, menopausal status, oral contraceptive use, hormone replacement therapy, alcohol intake, physical activity, smoking status, statins, low dose aspirin, Charlson comorbidity index, benign breast disease, family history of breast cancer) that could explain some of the missing data.
