# Supplementary figures and images for: Long-term exposure to insulin and volumetric mammographic density: observational and genetic associations in the Karma study
Source: Breast Cancer Res. 2018 Aug 9;20:93. doi: 10.1186/s13058-018-1026-7 (PMC6085687; doi:10.1186/s13058-018-1026-7)

Percent dense volume

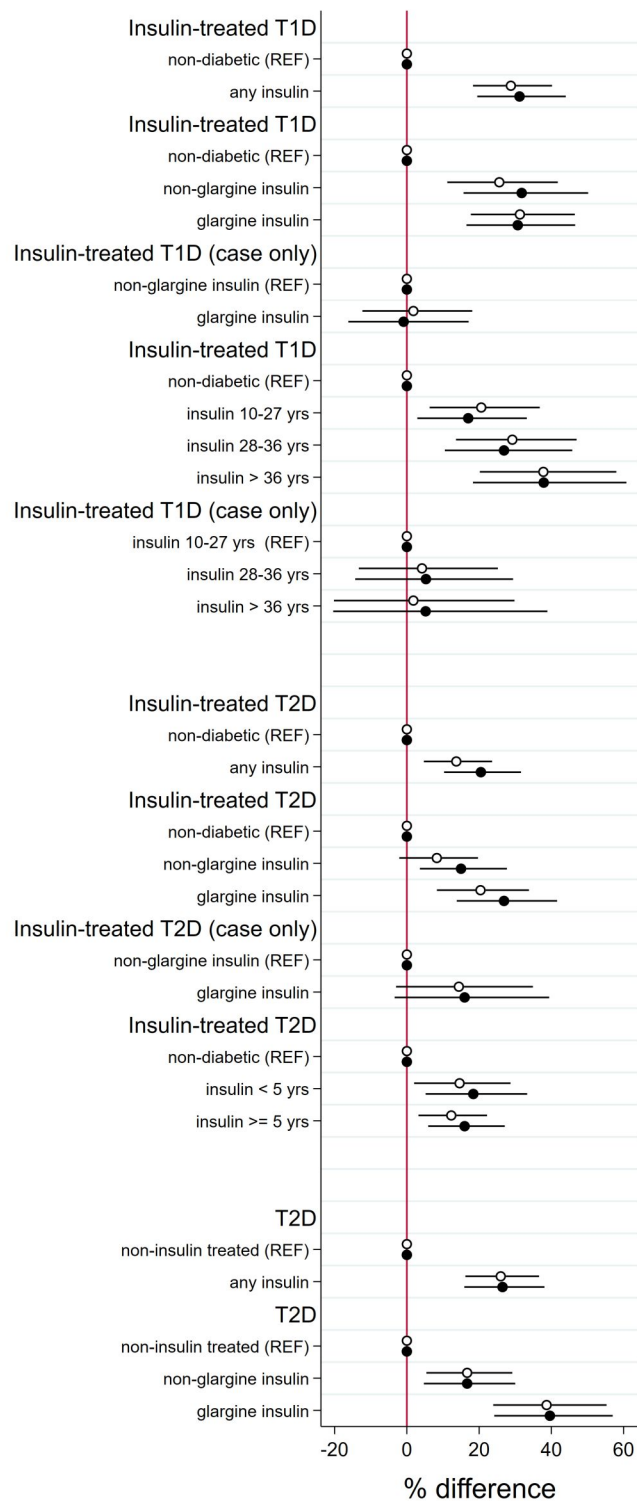

Absolute dense volume

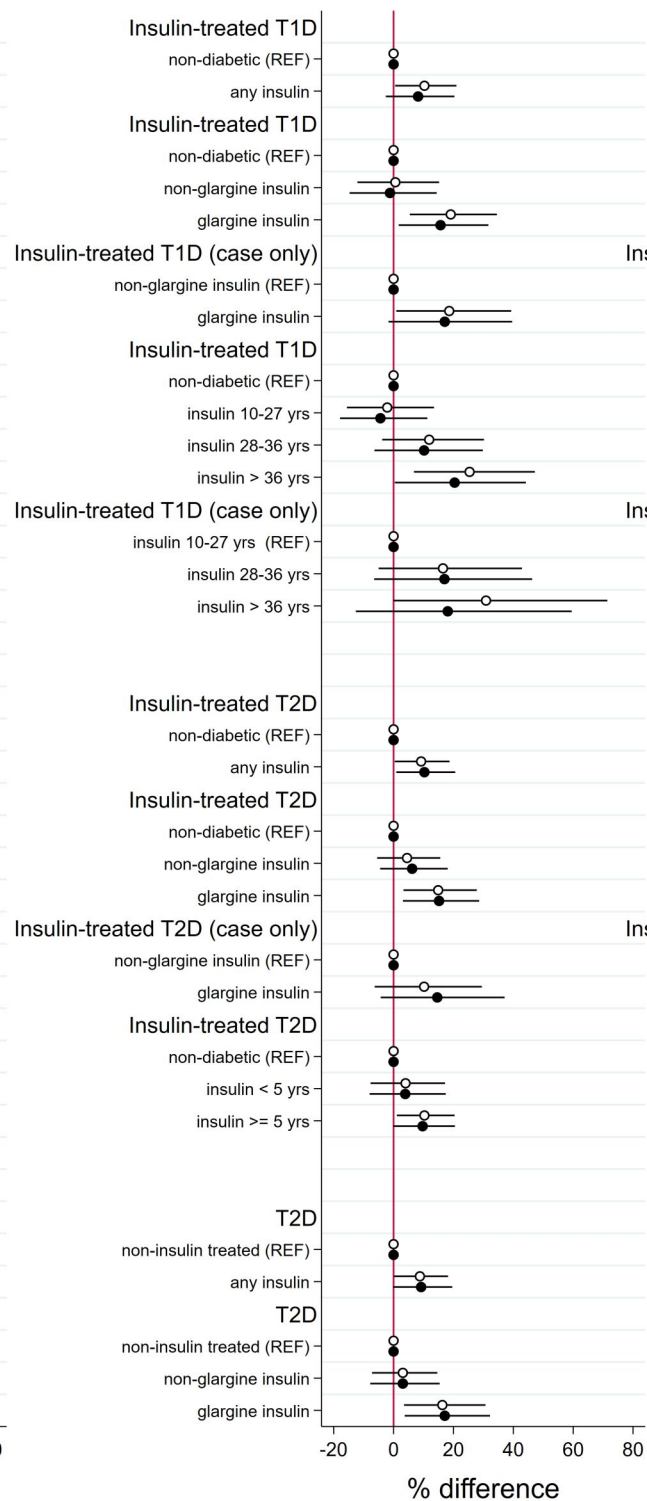

Absolute non-dense volume

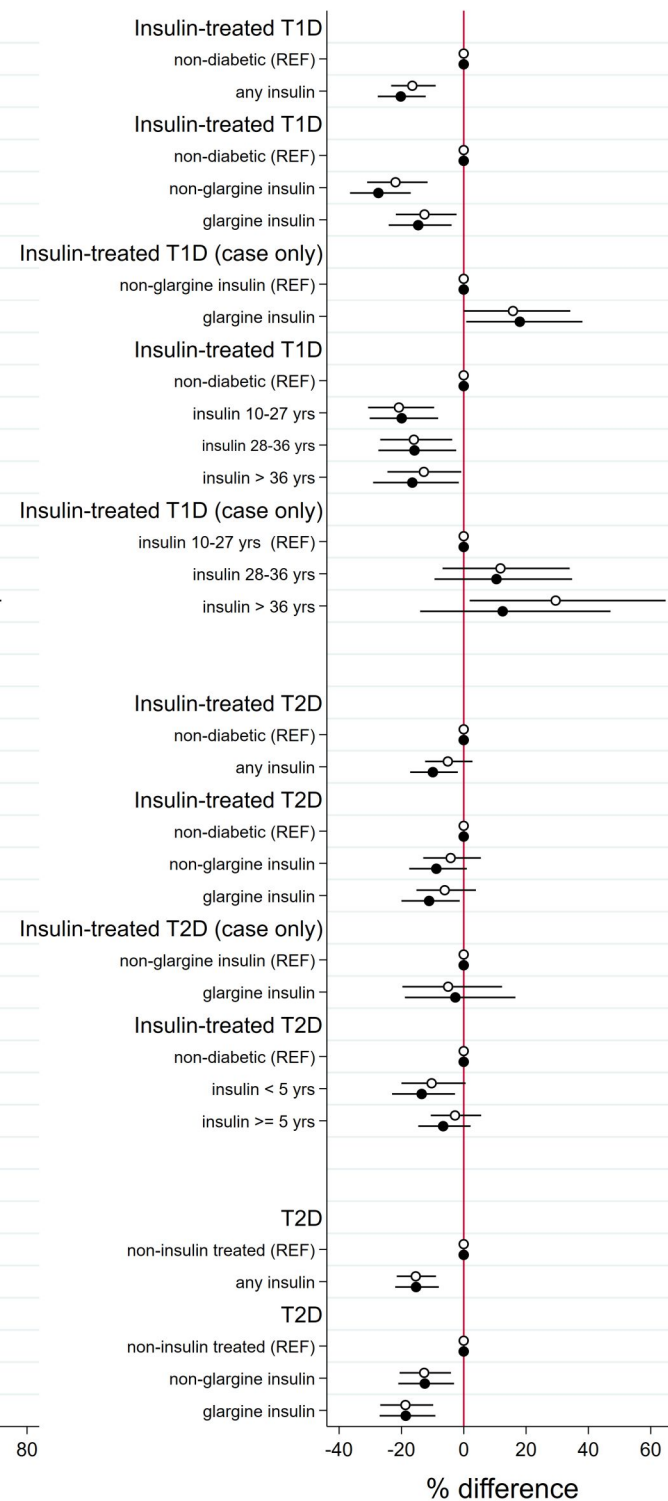

Supplement: Supplementary file 3 — Figure S1. Summary of percentage differences in volumetric mammography density observed by insulin therapy across the different observational analyses. Association of insulin therapy with volumetric mammographic density in different observational analyses with age and BMI adjusted estimates (open circles) and multivariable adjusted estimates (closed circles). Betas represent % differences in percent dense, absolute dense and absolute non-dense volume for differences in insulin exposure. (PDF 476 kb) [file 13058_2018_1026_MOESM3_ESM.pdf]

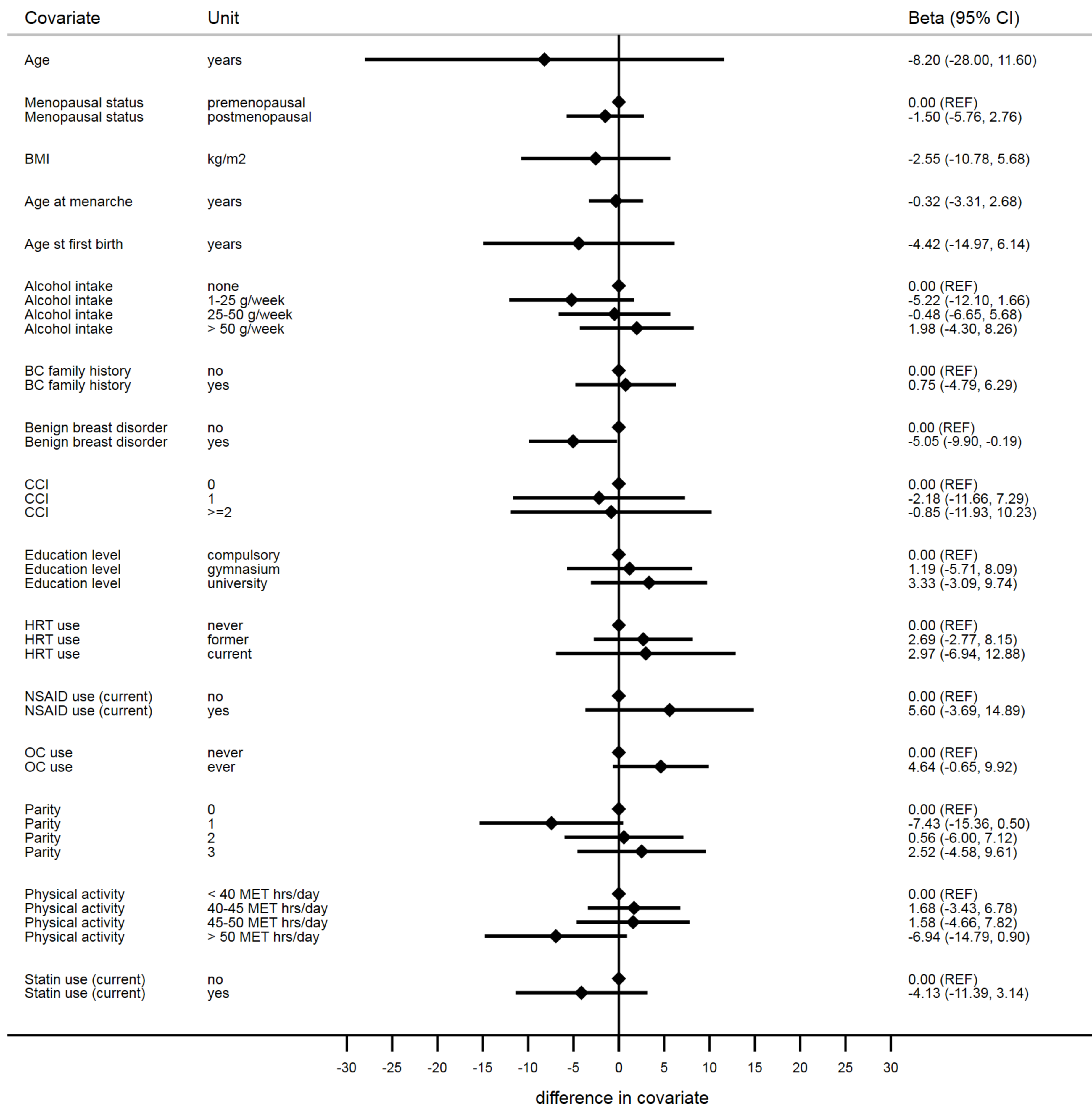

Supplement: Supplementary file 4 — Figure S2. Association of the insulin genetic score with potential confounders. Association of 18-SNP insulin genetic score with potential confounders in Karma sub-cohort of non-diabetic women with genotyping data. Associations were tested using linear regression and (multinomial) logistic regression, adjusting for age, six principal components and genotyping array. Betas represent differences in covariate level per 1-standard deviation increment in insulin genetic score. (PDF 225 kb) [file 13058_2018_1026_MOESM4_ESM.pdf]

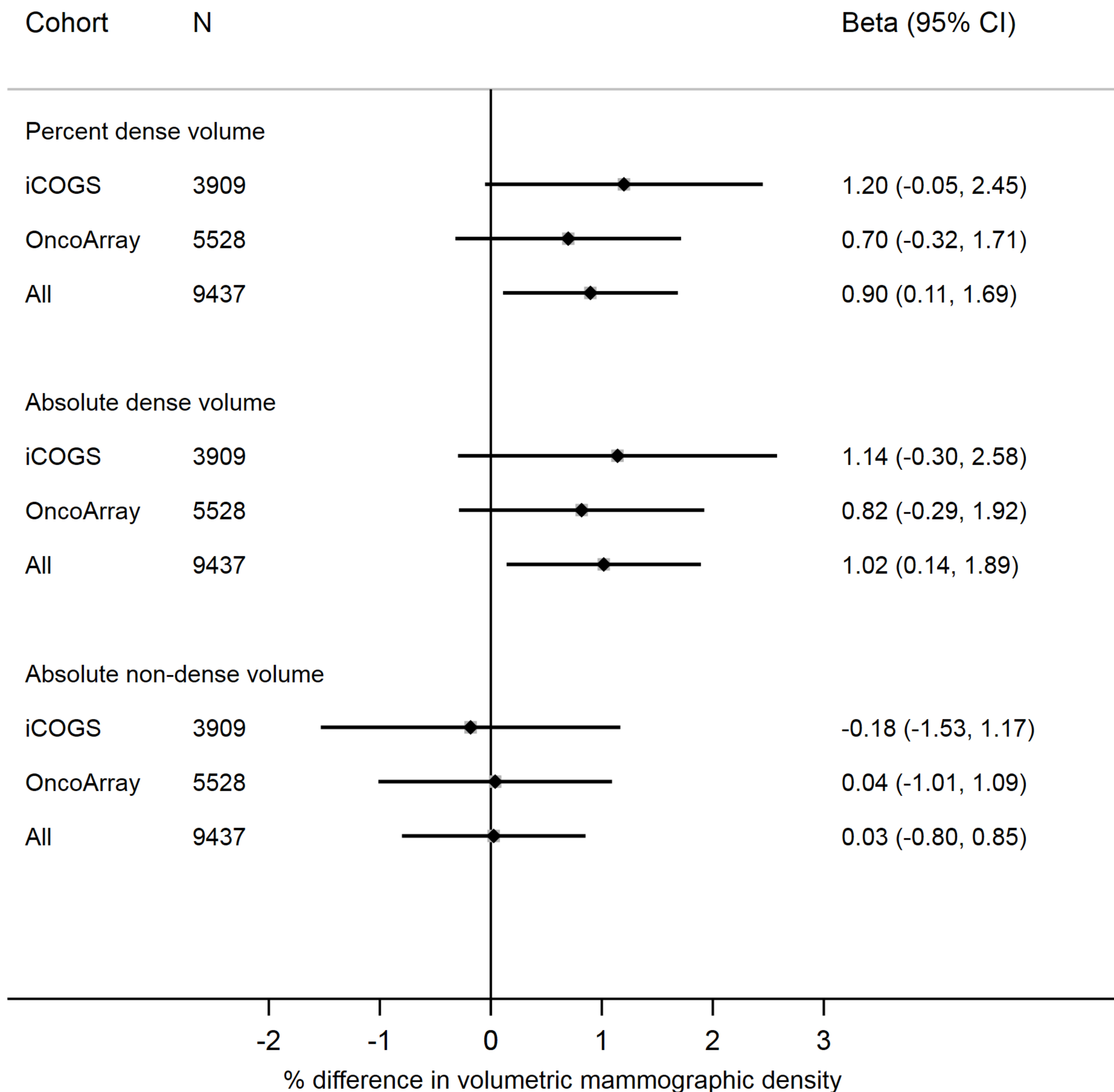

Supplement: Supplementary file 5 — Figure S3. Association of the insulin genetic score with volumetric mammographic density measures after additional adjustment for benign breast disease. Association of 18-SNP insulin genetic score with volumetric mammographic density in Karma sub-cohort of non-diabetic women with genotyping data, overall and stratified by genotyping array and with additional adjustment for benign breast disease. Associations with volumetric mammographic density were tested by linear regression, adjusting for age, body mass index, menopausal status, six principal components and benign breast disease. Analyses in total sub-cohort were additionally adjusted for genotyping array. All volumetric mammographic density measures were log-transformed prior to analyses, with betas representing % differences in volumetric mammographic density per 1-standard deviation increment in insulin genetic score. (PDF 146 kb) [file 13058_2018_1026_MOESM5_ESM.pdf]
